# IRF1 regulation of ZBP1 links mitochondrial DNA and chondrocyte damage in osteoarthritis

**DOI:** 10.1186/s12964-024-01744-1

**Published:** 2024-07-18

**Authors:** Kai Sun, Fan Lu, Liangcai Hou, Xiong Zhang, Chunran Pan, Haigang Liu, Zehang Zheng, Zhou Guo, Zhaoxuan Ruan, Yanjun Hou, Jinming Zhang, Fengjing Guo, Wentao Zhu

**Affiliations:** Department of Orthopedics, Tongji Hospital, Tongji Medical College, Huazhong University of Science and Technology, Wuhan, Hubei 430030 China

**Keywords:** Osteoarthritis, Chondrocyte, ZBP1, mtDNA, IRF1

## Abstract

**Background:**

Z-DNA binding protein 1 (ZBP1) is a nucleic acid sensor that is involved in multiple inflammatory diseases, but whether and how it contributes to osteoarthritis (OA) are unclear.

**Methods:**

Cartilage tissues were harvested from patients with OA and a murine model of OA to evaluate ZBP1 expression. Subsequently, the functional role and mechanism of ZBP1 were examined in primary chondrocytes, and the role of ZBP1 in OA was explored in mouse models.

**Results:**

We showed the upregulation of ZBP1 in articular cartilage originating from OA patients and mice with OA after destabilization of the medial meniscus (DMM) surgery. Specifically, knockdown of ZBP1 alleviated chondrocyte damage and protected mice from DMM-induced OA. Mechanistically, tumor necrosis factor alpha induced ZBP1 overexpression in an interferon regulatory factor 1 (IRF1)-dependent manner and elicited the activation of ZBP1 via mitochondrial DNA (mtDNA) release and ZBP1 binding. The upregulated and activated ZBP1 could interact with receptor-interacting protein kinase 1 and activate the transforming growth factor-beta-activated kinase 1-NF-κB signaling pathway, which led to chondrocyte inflammation and extracellular matrix degradation. Moreover, inhibition of the mtDNA-IRF1-ZBP1 axis with Cyclosporine A, a blocker of mtDNA release, could delay the progression of DMM-induced OA.

**Conclusions:**

Our data revealed the pathological role of the mtDNA-IRF1-ZBP1 axis in OA chondrocytes, suggesting that inhibition of this axis could be a viable therapeutic approach for OA.

**Supplementary Information:**

The online version contains supplementary material available at 10.1186/s12964-024-01744-1.

## Background

Osteoarthritis (OA) is one of the most common degenerative diseases, affecting more than 500 million people worldwide [1]. The most basic pathological feature of OA is the destruction of articular cartilage [1]. Chondrocytes, the predominant cell type in articular cartilage, govern the integrity and homeostasis of cartilage [2]. However, the molecular mechanisms underlying OA chondrocyte damage and OA pathogenesis are largely unknown. This gap prevents the development of effective agents to delay OA progression. Therefore, novel targets for OA treatment must be identified.

Innate immune sensing of pathogen-associated or damage-associated molecular patterns (PAMPs and DAMPs) can activate multiple intracellular signaling pathways that elicit a proinflammatory immune response [3]. In addition, many innate sensors also trigger inflammation in response to pathogens or other immunological stimuli [4].

These molecules, either from the extracellular matrix (ECM) or from dying cells, bind to pathogen-recognition receptors, such as Toll-like receptors and the receptor for advanced glycation end products, and activate the secretion of proinflammatory factors, leading to joint inflammation [5]. At the cellular level, these molecules could cause the chondrocyte damage including imbalanced ECM metabolism, inflammation, and death in chondrocytes [6, 7].

Recent studies have identified Z-DNA binding protein 1 (ZBP1) as an innate sensor of viral infections and endogenous nucleic acid ligands such as mitochondrial DNA (mtDNA), regulating cell death, inflammasome activation, and inflammatory responses [8–10]. Given the critical role of ZBP1 as a pathogen sensor and a regulator of cell death and inflammation, ZBP1 is also involved in several diseases, such as infection [11], skin inflammation [12], and inflammatory bowel disease [13]. Although PAMP- and DAMP-associated inflammation play roles in chondrocyte damage and OA progression [14, 15], whether ZBP1 contributes to the development of OA remains poorly understood.

Under physiological conditions, DNA mainly resides in the nucleus and mitochondria of cells. Damaged or stressed mtDNA can be released into the cytosol through pores formed by Voltage-Dependent Anion Channel (VDAC) oligomers or the mitochondrial permeability transition pore (mPTP) [16, 17]. Numerous studies have reported that the release of mtDNA is involved in the pathogenesis of various diseases. Szczesny et al. reported that activation of the mtDNA/ZBP1 pathway by oxidative stress triggers the expression of proinflammatory markers in retinal pigment epithelial cells and contributes to the pathogenesis of age-related macular degeneration [18]. The mitochondrial DNA concentration in synovial fluid reflects the degree of cartilage damage [19], suggesting a close correlation between mtDNA and OA pathology.

IFN regulatory factor (IRF)1 is a transcription factor with a central role in innate and adaptive immune responses [20]. Recent studies have reported that IRF1 expression is significantly elevated after mtDNA stimulation in retinal microvascular endothelial cells [21] and that IRF1 can mediate ZBP1 transcription in trophoblast cells [22]. These findings establish correlations between mtDNA, IRF1, and ZBP1; however, the role of the mtDNA-IRF1-ZBP1 axis in chondrocyte damage and OA is largely unknown.

In this study, we revealed a positive correlation between ZBP1 expression and OA progression. We also identified mtDNA-IRF1-ZBP1-RIPK1 as the pathological axis involved in chondrocyte damage. In addition, this study demonstrated that inhibition of the mtDNA-IRF1-ZBP1 axis with Cyclosporine A (CsA) protected mice from destabilization of the medial meniscus (DMM)-induced OA progression.

## Methods

### Human samples

All OA cartilage specimens were collected from patients who underwent surgery to replace the knee joint at Tongji Hospital. The control cartilage was obtained from patients who underwent amputation surgery. The informed consent forms for cartilage specimen collection were signed by the patients and the collection of and experiments using human cartilage were approved by the Ethics Committee of Tongji Hospital (TJ-IRB20210905). The basic information of the amputees and the OA patients is shown in Additional file 1: Tables S1-2.

### Materials and reagents

The following reagents were obtained commercially: tumor necrosis factor-α (TNF-a) was obtained from R&D systems (Minneapolis, MN, USA; #554,589). IL-1β was obtained from R&D Systems (Minneapolis, MN, USA; 401-ML-010). Cyclosporin A (CsA) was obtained from MedChemExpress (New Jersey, USA; HY-B0579). Fetal bovine serum was obtained from NEW-ZEALAND (New York, USA). iNOS Rabbit mAb (13,120; Western blot: 1:1000), COX2 Rabbit mAb (12,882; Western blot: 1:1000) and Phospho-NF-κB p65 (Ser536) Rabbit mAb (3033; Western blot: 1:1000; IF:1:800) were purchased from Cell Signaling Technology, Inc. (Beverly, USA). dsDNA Mouse mAb (ab27156; Western blot: 1:1000; IF: 1:600) antibodies were obtained from Abcam (Cambridge, UK). COL2A1 Rabbit Polyclonal antibody (28459-1-AP; Western blot: 1:1000; IF: 1:200; IHC: 1:800), β-ACTIN Recombinant Rabbit antibody (81115-1-RR; Western blot: 1:10000), Phospho-RIPK1 (Ser161) Mouse Monoclonal antibody (66854-1-Ig; Western blot: 1:5000; IF: 1:400; IHC: 1:500), IRF1 Rabbit Polyclonal antibody (11335-1-AP; Western blot: 1:500; IF: 1:250), GAPDH Mouse mAb (60004-1-Ig; Western blot: 1:10000) and MMP13 Rabbit Polyclonal antibody (18165-1-AP; Western blot: 1:1000; IF: 1:200; IHC: 1:100) were acquired from Proteintech Group (Wuhan, China). The ZBP1 Rabbit Polyclonal Antibody was obtained from Thermo Fisher (Massachusetts, USA, PA5-20455; Western blot: 1:1000; IF: 1:200; IHC: 1:500). MMP3 Rabbit monoclonal antibody (BM4074; Western blot: 1:1000), FITC-conjugated goat anti-mouse and anti-rabbit secondary antibodies, type II collagenase, Dulbecco’s modified Eagle’s culture medium F12 (DMEM/F12) and trypsin were purchased from Boster (Wuhan, China). IP lysis buffer (p0013) and MitoTracker were obtained from Beyotime (Shanghai, China).

### Chondrocyte isolation and culture

Primary chondrocytes were isolated from the knee joints of 5-day-old house mice. First, the cartilage was dissected into pieces in sterilized phosphate-buffered saline (PBS), and the cartilage slices were subsequently digested with 0.25% trypsin at 37 °C for 30 min. Then,, the cartilage was digested with 0.2% type II collagenase in DMEM for 6 h. Afterward, the cell suspension was transferred to a centrifuge tube and centrifuged at 1,200-1,800 rpm at room temperature for 5 min. Primary chondrocytes were resuspended and cultured in DMEM/F12 containing 10% fetal bovine serum. Next, the chondrocytes were passaged in a T25 flask for culture, and the medium was changed every 2 days. Only the first or second passages of chondrocytes were used in the experiments.

### Small interfering RNAs (siRNAs), plasmids and transfection

The negative control siRNA and siRNAs targeting ZBP1 and IRF1 were synthesized by Tsingke Biotechnology. The transfection procedure was conducted as previously described [23]. After the chondrocytes had grown to 60–70% confluence on six-well plates, they were transfected with 50 nmol of the negative control small interfering RNA (siNC), ZBP1 siRNA (siZBP1 #1: CCCTCAATCAAGTCCTTTA; siZBP1 #2: GCCTGCAACATGGAGCATA; siZBP1 #3: CCTGTATTCCATGAGAAAT), or IRF1 siRNA (siIRF1 #1: CACTGATCTGTATAACCTA; siIRF1 #2: GATGGACATTATACCAGAT; siIRF1 #3: GCAGATGGACATTATACCA) using Lipofectamine 3000 in accordance with the manufacturer’s protocol. Then, the chondrocytes were treated with IL-1β or TNF-α before total RNA and protein extraction. Western blot and qRT‒PCR were performed to assess the knockdown efficiency compared to that in the siNC group. The negative control plasmid and flag-tagged ZBP1 plasmid were synthesized by Aoke Biosciences.

### The RHIM domain of ZBP1 was mutated and loaded in an adenovirus

Recombinant lentivirus vectors which expressing ZBP1 or ZBP1 which mutant the RHIM domain were constructed by DesignGene (Wuhan, China). Briefly, DNA fragments encoding ZBP1 or ZBP1 which mutant the RHIM domain were subcloned into the lentivirus vectors (NM_021394.2). The empty lentivirus vector was used as a negative control. Subsequently, chondrocytes were cultured in the 10 cm dish, and transfected with lentivirus when the cell density reached to about 80%. The transfect time duration was 24 h. Then, the chondrocytes were cultured useing the fresh medium for another 24 h. Finally, the chondrocytes were treated with TNF-α for 15 min. Chondrocytes were infected with lentivirus at 30 multiplicities of infection (MOI).

### In vivo downregulation of ZBP1 using an adeno-associated virus

Adeno-associated virus 9 containing ZBP1 (AAV9-shZBP1) (WZ Biosciences, Inc.) was administered to eight-week-old C57BL/6J mice by intra-articular injection. The doses (10 µl, 1 × 10^12^ vg/ml) used for the joint injection of AAV9 were chosen according to a previous report [24]. Three weeks after the injection of adeno-associated virus into the joint cavity, destabilization of the medial meniscus (DMM) surgery was performed to establish the animal model. At the eighth week after surgery, the knee joints of the mice were collected for subsequent experiments. Other groups that did not require an injection of AAV9-shZBP1 were treated with a negative control (AAV9-GFP) for the same time.

### Animal experiments

Eight-week-old male C57BL/6 mice were used for the animal experiments. The in vivo study was approved by the Institutional Animal Care and Use Committee of Tongji Hospital (IACUC No.: TJH202301008). DMM surgery was conducted according to previously described instructions [25] to induce posttraumatic OA when the C57BL/6 mice were anesthetized. Mice in the control group underwent a sham operation via an incision in the medial capsule of the knee joint. Thirty-two male C57BL/6 mice were indiscriminately separated into four groups: Sham + AAV9-GFP, Sham + AAV9-shZBP1, DMM + AAV9-GFP, and DMM + AAV9-shZBP1. For knee joint capsule administration of CsA, we indiscriminately separated thirty mice into three groups: DMM + PBS, DMM + CsA (2 µg/kg), and DMM + CsA (20 µg/kg). Then, 10 µl of CsA or PBS was injected into the knee cavity once a week for seven weeks. The mice were sacrificed eight weeks after DMM or Sham surgery.

### Western blot analysis

The treated chondrocytes were lysed with RIPA buffer (Biocolors Biotechnology Co., Shanghai, China), and the samples were sonicated 3 times. Then, the samples were centrifuged at 12,000 rpm for 30 min at 4 °C. The supernatant was quantified using the bicinchoninic acid method. Next, the samples were heated with loading buffer at 95 °C for 10 min. The 25 µg protein samples were separated by SDS-PAGE and transferred to polyvinyl difluoride membranes. Afterward, the PVDF membranes were blocked with blocking solution (5% skim milk) for 1 h at room temperature and then incubated with primary antibodies against MMP13, MMP3, GAPDH, COL2A1, IRF1, COX2, AGGRECAN, iNOS, SOX9, ZBP1, β-actin, P65, P-P65, TAK1, P-TAK1, RIPK1, and P-RIPK1 at 4 °C. After an overnight incubation, the membranes were washed with Tris-buffered saline with Tween (TBST) four times for 7 min. The next step was to incubate the membranes with the secondary antibody, after which the membranes were washed as described above. Finally, the membranes were subjected to chemiluminescence. We selected GAPDH or β-actin as the reference. A Bio-Rad scanner was used to detect the signal strength on the membranes.

### Quantitative real-time PCR

A total RNA extraction kit (Omega Biotek, R6834-01) was used to extract the RNA for qPCR. Complementary DNA was synthesized using Hifair^®^ III 1st Strand cDNA Synthesis SuperMix (Yeasen, 11141ES60). Then, the cDNA was amplified using SYBR Green Master Mix (Yeasen, 11203ES03). The amplification method was described previously. *Gapdh* served as a reference. All mRNA expression levels were standardized to *Gapdh*. The primer sequences utilized in the study were *Zbp1* (F) 5’-AAGAGTCCCCTGCGATTATTTG-3’ and (R) 5’-TCTGGATGGCGTTTGAATTGG-3’; *Irf1* (F) 5’-ATGCCAATCACTCGAATGCG-3’, (R) 5’-TTGTATCGGCCTG TGTGAATG-3’; *Inos* (F) 5’-GTTCTCAGCCCAACAATAC AAGA-3’, (R) 5’-GTGGACGGGTCGATGTCAC-3’; and *Gapdh* (F) 5’-AGGTTGTCTCCTGCGACTT CA-3’, (R) 5’-GGGTG GTCCAGGGTTTCTTA-3’. The tests were repeated 3 times.

### Detection of mtDNA in cytosolic extracts

mtDNA release was assayed as described previously [25, 26]. The cell fraction was collected as described previously [26, 27]. Briefly, chondrocytes were washed with PBS, 10% of which was used to extract DNA from the entire cell. The other chondrocytes were resuspended in precooled mitochondrial extraction buffer 1 (70 mM sucrose, 2 mg/ml bovine serum albumin, 20 mM HEPES-KOH, 220 mM mannitol, pH 7.5, and 1 mM EDTA) supplemented with a protease inhibitor cocktail and a phosphatase inhibitor. Next, the homogenized cells were centrifuged for 15 minutes (1000 × g, 4°C). The supernatant was centrifuged again at 10,000 × g for 10 minutes at 4°C to pellet the mitochondria from the supernatant of the cytosolic fraction. One part was extracted using a DNA extraction kit (Tiangen Biotech, China), and the extracts were used as standardization controls for total DNA. The other sample was centrifuged to obtain pure cytosolic fractions. DNA was subsequently extracted from the fractions via QIAQuick Nucleotide Removal Columns (QIAGEN, Germany). Then, the total DNA and mitochondrial DNA were amplified using qPCR. The Ct value for the mtDNA number in the whole-cell extracts was regarded as a standardization control for comparing the variation in mtDNA levels in the cytosol. The presence of TERT DNA indicated that nuclear lysis did not occur. The sequences of the primers used in this experiment are *D-loop* (F) 5’-AATCTACCATCCTCCGTGAAACC-3’ and (R) 5’-TCAGTTTAGCTACCCCCAAGTTTAA-3’; *Loop2* (F) 5’-CCCTT CCCCATTTGGTCT-3’, (R) 5’-TGGTTTCACGGAGGATGG-3’; *Loop3* (F) 5’-TCCTCCGTGAAACCAACAA − 3’, (R) 5’-AGCGAGAAGAGGGGCATT-3’; and TERT (F) 5’-CTAGCTCATGTGTCAAGACCCTCTT-3’, (R) 5’-GCCAGCACGTTTC TCTCGTT-3’.

### Coimmunoprecipitation (co-IP) assay

The co-IP assay was conducted as previously reported [28]. Briefly, chondrocytes were cultured and treated with TNF-α for 15 min. Afterward, the cells were lysed in 1 mL of IP lysis buffer supplemented with protease inhibitors. The samples were centrifuged at 4 °C for 15 min after being lysed on ice for 10 min. Protein A + G magnetic beads were added to the cell lysates, which were then shaken for 1 h to eliminate nonspecific binding. Subsequently, the mixture was shaken overnight at 4 °C after being mixed with the primary antibody. Next, the samples were centrifuged, the supernatant was removed, and the immune complex was washed 3 times with lysis buffer. Finally, lysis buffer and 5× loading buffer were added to the beads. After the mixture was boiled for 10 min at 97 °C, the supernatant was removed for western blot analysis.

### Immunofluorescence (IF) staining

First, chondrocytes were seeded in confocal dishes, and the expression of ZBP1 or IRF1 was knocked down according to previous methods. Next, the cells were treated with or without TNF-α for a suitable time period. Then, the chondrocytes were fixed with 4% paraformaldehyde for 10 min, and 0.5% Triton X-100 was used to permeabilize the cell membrane. Afterward, the chondrocytes were incubated with blocking goat serum for 1 h, after which they were incubated with primary antibodies against MMP13, COL2A1, ZBP1, P-P65, P-RIPK1, and double-stranded DNA (dsDNA), as well as the corresponding secondary antibodies. After the incubations, the nuclei were stained with DAPI for 3 min. Next, the cells were washed with PBST between each step. A laser scanning confocal microscope (FV3000, Olympus Corporation, Japan) was used to capture the images.

### Histological and immunohistochemical (IHC) analyses

Mouse knee joints were fixed with paraformaldehyde for 1 day. The samples were decalcified for 21 days with 10% EDTA (pH 7.4) and cut into 5-µm thick sagittal sections after they were embedded in paraffin. Next, the samples were stained with safranin O/fast green and hematoxylin–eosin (HE). The OARSI histopathology scoring system was applied in a blinded manner to evaluate cartilage degeneration. After the samples were deparaffinized and rehydrated, BSA containing 0.1% Triton X-100 was applied to block the samples for 1 h. Next, the samples were incubated with anti-COL2A1, anti-P-P65, anti-P-RIPK1, anti-MMP13 or anti-ZBP1 antibodies, incubated with the secondary antibody, and stained with DAB. Finally, hematoxylin was used to counterstain the samples. Images were obtained using a general microscope (BX53, Olympus Corporation, Japan).

### Microcomputed tomography (micro-CT) analysis

First, the joints were evaluated with a Viva CT 80 scanner (Scanco Medical AG, Switzerland), and the samples were scanned at a thickness of 10.5 μm. Images of reconstructed 3-dimensional (3D) regions were obtained using built-in software. The dimensions of the tibial plateau osteophytes were detected and quantified in cross-sectional images. The analysis of subchondral bone in the medial condyle of the tibia began at the distal 10th layer of the tibial plateau superior border and ended at the 40th layer. Morphological parameters, such as the bone volume/tissue volume (BV/TV), the number of trabeculae (Tb.N), the trabecular space (Tb.Sp) and the thickness of the trabecula (Tb.Th), were analyzed.

### Statistical analysis

The unpaired two-tailed Student’s t test was used to compare two different groups, and one-way ANOVA followed by Tukey’s post hoc test and two-way ANOVA followed by Sidak’s multiple comparisons test for multiple comparisons. A. The data are presented as the means ± standard deviations (SDs). At least three biological replicates were analyzed in each experiment. The statistical analysis was performed with GraphPad Prism 9.0. *P* < 0.05 was regarded as significant, NS represents not significant, * indicates *P* < 0.05, and ** indicates *P* < 0.01.

## Results

### OA features by the altered ZBP1 expression

**Fig. 1:**
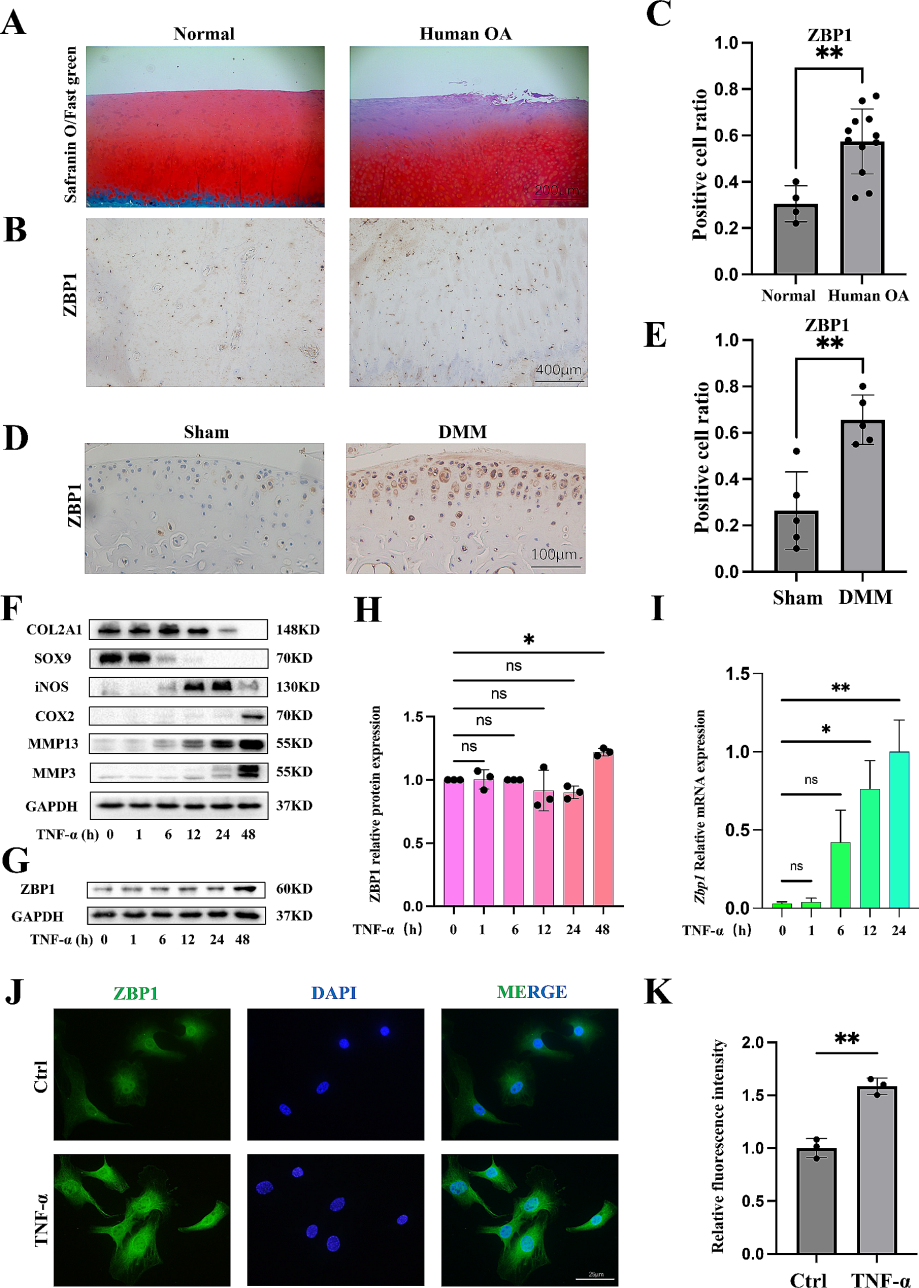
ZBP1 expression in OA. (A, B) Safranin O/fast green and IHC staining of ZBP1 in normal cartilage and human OA cartilage (scale bar A: 200 μm, scale bar B: 400 μm). (C) Statistical analysis of ZBP1-positive chondrocytes among all chondrocytes (control group: *n* = 4; human OA group: *n* = 12). (D, E) IHC images and quantitative analysis of ZBP1-positive cells in the knee joint cartilage of mice (sham group: *n* = 5; DMM group: *n* = 5; scale bar: 100 μm). (F) Western blot analysis of the levels of the indicated proteins in chondrocytes treated with TNF-α for 0, 1, 6, 12, 24 and 48 h. (G, H) Western blot and semiquantitative analysis of ZBP1 protein expression (*n* = 3). (I) qPCR results showing the relative ZBP1 expression level in chondrocytes treated with TNF-α for 0, 1, 6, 12, or 24 h (*n* = 3). (J, K) Image of immunofluorescence staining and quantitative analysis of the fluorescence intensity showing the relative expression of ZBP1 in chondrocytes treated with TNF-α for 24 h (*n* = 3; scale bar: 25 μm). The data are shown as the means ± SDs. *P < 0.05, **P < 0.01

We first assessed the ZBP1 expression level in OA samples and a posttraumatic OA murine model to determine the relationship between ZBP1 expression and OA progression. Severe degeneration was observed in the cartilage of OA patients, and the IHC results indicated that more ZBP1-expressing chondrocytes were found in the cartilage of OA patients than in that of normal controls (Fig. 1A-C). We established a posttraumatic OA model by performing DMM surgery, and the IHC results for ZBP1 expression in the mouse joint cartilage were consistent with the results from human samples (Fig. 1D, E). Subsequently, we detected the expression of ZBP1 in vitro. The pathogenesis of OA is closely related to proinflammatory factors [29]. Therefore, we applied TNF-α to mimic pathological conditions within OA joints and found that TNF-α caused increased expression of inflammatory mediators (iNOS and COX2) and matrix-degrading enzymes (MMP3 and MMP13) while decreased matrix-synthesis markers (COL2A1 and SOX9) (Fig. 1F). The Western blot and qPCR results showed that the ZBP1 level was notably increased in chondrocytes after TNF-α treatment (Fig. 1G-I). Consistent with these results, IF staining confirmed a distinct increase in ZBP1 expression in chondrocytes treated with TNF-α (Fig. 1J, K). Collectively, these data support the hypothesis that OA induces ZBP1 overexpression.

### ZBP1 is required for chondrocyte inflammation and ECM degradation

**Fig. 2:**
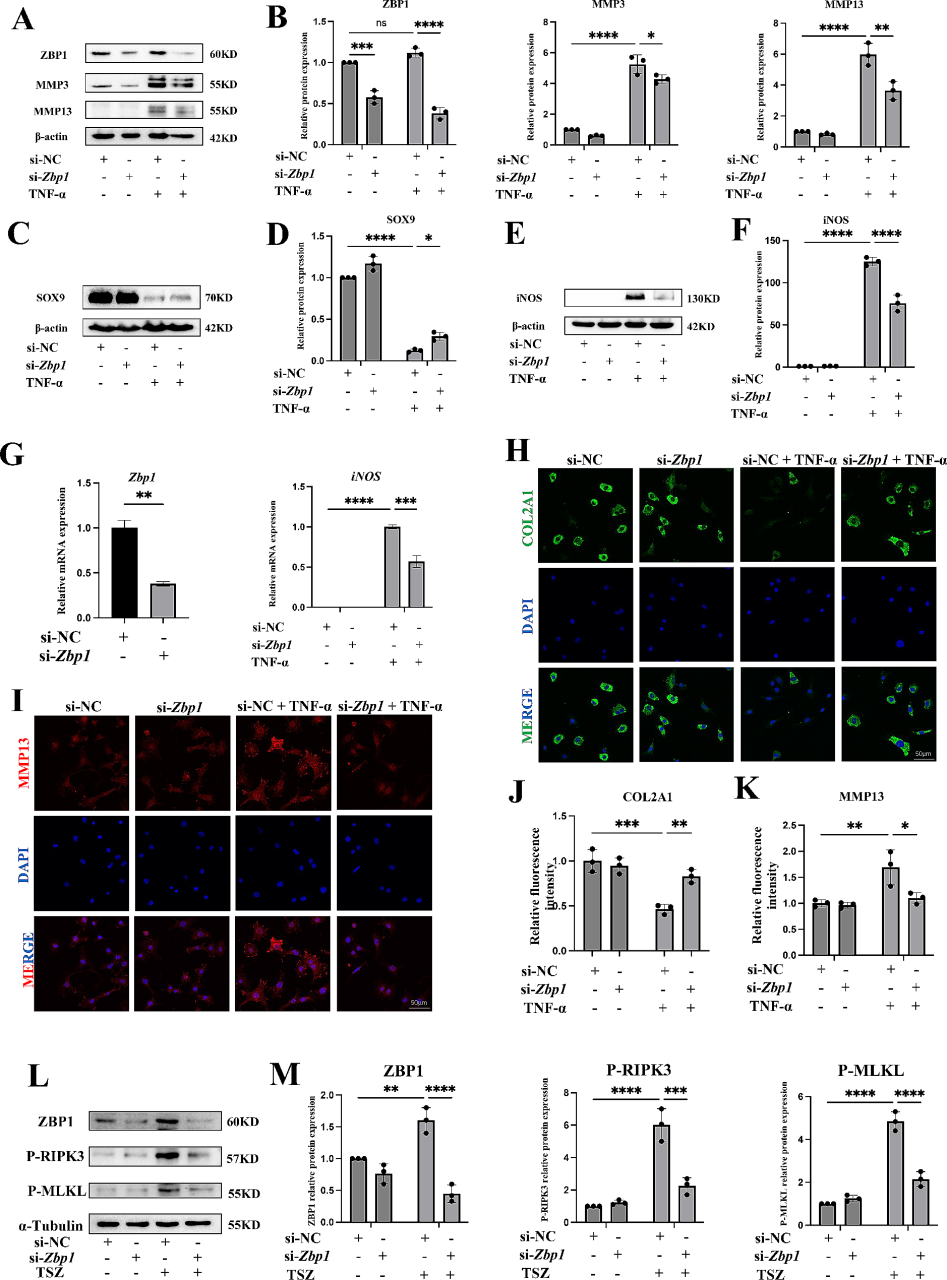
ZBP1 is essential for chondrocyte damage. Chondrocytes were transfected with siNC or ZBP1 siRNA for 24 h following TNF-α induction for 24 h. (A, B) Western blots and quantitative analysis of ZBP1, MMP13, and MMP3 expression levels (*n* = 3). (C, D) Western blots and quantitative analysis of SOX9 expression levels. (E, F) Western blot and quantitative analysis of iNOS expression levels (*n* = 3). (G) qPCR results showing the relative expression levels of *Zbp1* and *Inos* (*n* = 3). (H-K) Immunofluorescence staining and fluorescence intensity analysis of the relative expression levels of COL2A1 and MMP13 (*n* = 3; scale bar: 50 μm). Chondrocytes were transfected with siNC or ZBP1 siRNA following TSZ (20 ng/ml TNF-α, 100 nM Smac mimetic, and 20 mM Z-VAD) induction for 12 h. (L, M) Western blots and quantitative analysis of ZBP1, P-RIPK3, and P-MLKL expression levels (*n* = 3). The data are shown as the means ± SDs. *P < 0.05, **P < 0.01, *** *P* < 0.001, **** *P* < 0.0001

We investigated the effect of ZBP1 knockdown on inflammatory chondrocytes to assess the functional relevance of ZBP1 in chondrocytes. First, the efficiency of the siRNA-mediated knockdown of ZBP1 expression was confirmed by the qPCR and Western blot results (Fig. 2A and Additional file 1: Fig. S1A). The most important function of chondrocytes is to maintain ECM homeostasis via anabolism and catabolism [30]. Therefore, we further detected changes in ECM metabolism using western blotting. ZBP1 knockdown significantly restored ECM homeostasis, as shown by reduced expression levels of the catabolic markers matrix metallopeptidase 13 (MMP13) and metallopeptidase 3 (MMP3) and increased expression level of the anabolic marker SOX9 (Fig. 2A-D). ZBP1 knockdown also markedly decreased the expression of inflammatory markers (iNOS; Fig. 2E, F). qPCR revealed similar results (Fig. 2G). In addition, the IF analysis confirmed that ZBP1 knockdown increased the COL2A1 expression level and decreased the MMP13 expression level (Fig. 2H-K). TSZ (20 ng/ml TNF-α (T), 100 nM Smac mimetic (S), and 20 mM Z-VAD (Z)) can induce necroptosis [30, 31]; therefore, we used TSZ to induce necroptosis in chondrocytes to verify the role of ZBP1 in this process. Western blot analysis revealed that TSZ increased the expression of ZBP1 and necroptosis markers, and knockdown of ZBP1 significantly decreased the expression of necroptosis markers (Fig. 2L, M). We also treated chondrocytes with interleukin-1β (IL-1β), another classic proinflammatory factor that is commonly used in OA studies, to determine the impact of ZBP1 on inflammatory chondrocytes. The Western blot and qPCR results showed that ZBP1 knockdown also attenuated IL-1β-induced chondrocyte inflammation and ECM degradation (Additional file 1: Fig. S2A, B). Collectively, these results indicated that ZBP1 contributed to the OA-like phenotype of chondrocytes.

### ZBP1 knockdown protects mice from DMM-induced cartilage degeneration and osteophyte formation

**Fig. 3:**
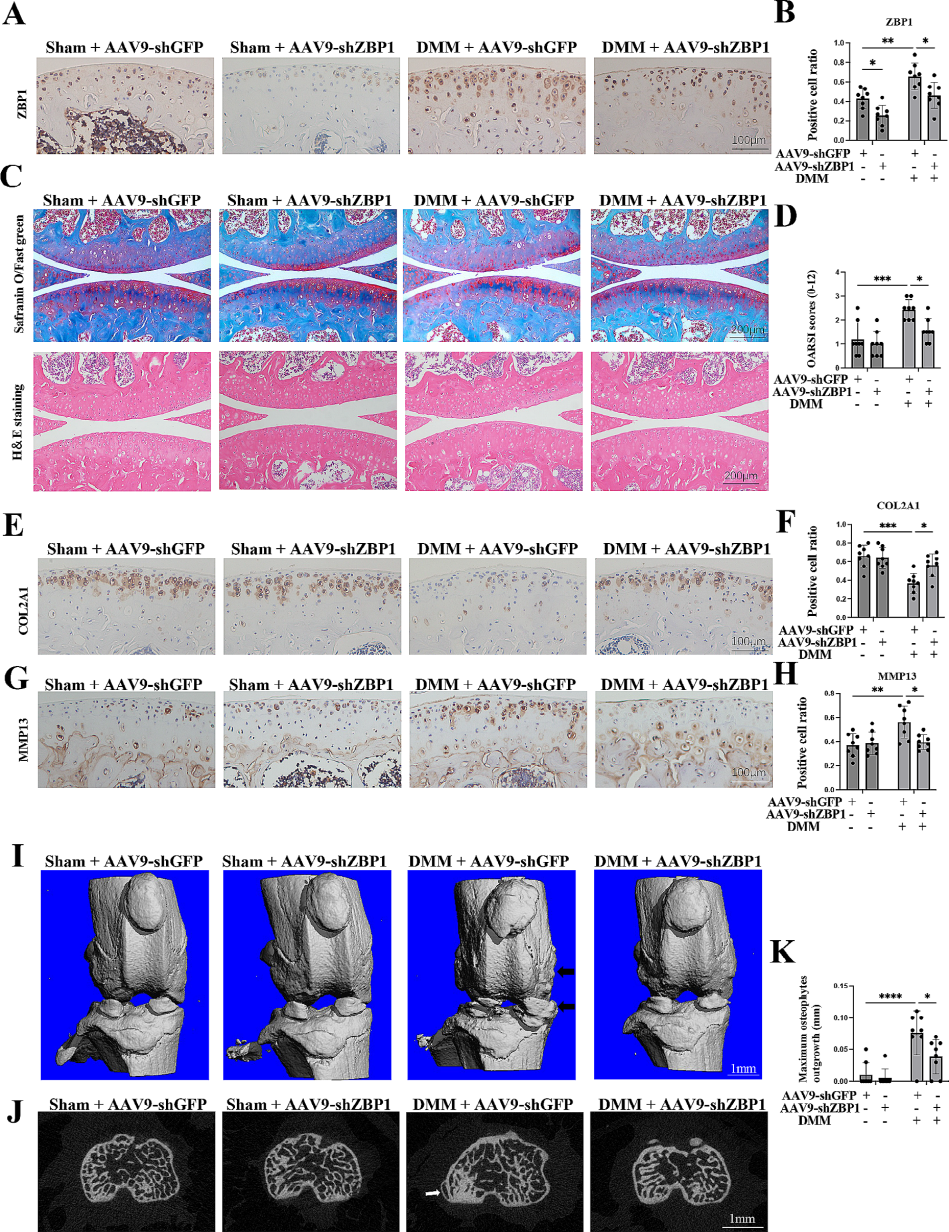
ZBP1 is essential for OA progression in DMM mice. (A, B) IHC staining showing ZBP1-positive cells in the cartilage of the four groups (scale bar: 100 μm). (C, D) Safranin O/fast green staining, H&E staining and OARSI scores of the four groups (sham + AAV9-shGFP, *n* = 8; sham + AAV9-shZBP1, *n* = 8; DMM + AAV9-shGFP, *n* = 8; DMM + AAV9-shZBP1, *n* = 8; scale bar: 200 μm). (E-H) IHC staining showing COL2A1-positive cells and MMP13-positive cells in the cartilage of the four groups (scale bar: 100 μm). (I) Images showing 3D reconstructions of mouse joints; the black arrow indicates an osteophyte (scale bar: 1 mm). (J, K) Transverse sectional images and statistical analysis of the maximum osteophyte outgrowth of each joint in the four groups; the white arrow indicates the osteophyte (scale bar: 1 mm). The data are shown as the means ± SDs. *P < 0.05, **P < 0.01, *** *P* < 0.001, **** *P* < 0.0001

Since ZBP1 is required for the OA-like phenotype of chondrocytes, the next key question is whether the loss of ZBP1 in chondrocytes protects mice from OA. AAV9-ZBP1 was injected into the knee cavity to downregulate ZBP1 expression in cartilage. The IHC results confirmed that AAV9-shZBP1 effectively decreased the number of ZBP1-chondrocytes compared to the AAV9-shGFP group (Fig. 3A, B).

Two weeks after the first injection of AAV9, the mice underwent either DMM surgery or a sham operation. Then, the mice were sacrificed on Day 56 following surgery. Safranin O/fast green staining revealed mild cartilage degradation, as evidenced by the loss of proteoglycans, cartilage erosion, and a higher OARSI score in the joints of the DMM group than in those of the sham group (Fig. 3C, D). In particular, compared with those in the DMM group, the mice in the AAV9-shZBP1 group exhibited less articular cartilage degeneration with lower OARSI scores (Fig. 3C, D). Consistently, the IHC analysis indicated significantly lower levels of MMP13 expression and higher levels of COL2A1 in the AAV-shZBP1-injected mice than in the DMM mice (Fig. 3E-H).

Subchondral bone remodeling and osteophyte formation are typical pathological features of OA [31–33]. Moreover, we evaluated the changes in subchondral bone and osteophytes in the four groups. AAV9-shZBP1-injected mice that underwent DMM surgery showed reduced osteophyte formation with a lower osteophyte score (Fig. 3I-K). However, no significant difference in subchondral bone was observed between the AAV9-shZBP1-treated group and the DMM group (Additional file 1: Fig. S3A). Taken together, these results suggested that the downregulation of ZBP1 in cartilage protected mice from DMM-induced cartilage degeneration and osteophyte formation.

### ZBP1 interacts with RIPK1 to promote chondrocyte damage via the NF-κB signaling pathway

As ZBP1 harbors the RIP homotypic interaction motif (RHIM), the interaction of ZBP1 with other RHIM-containing proteins is the foundation of ZBP1 signal transduction, and our previous study showed the pathological role of the RIPK1-NF-κB signaling pathway in inflammatory chondrocytes [34, 35], this evidence prompted us to analyze the interaction between ZBP1 and RIPK1 in chondrocytes.

**Fig. 4:**
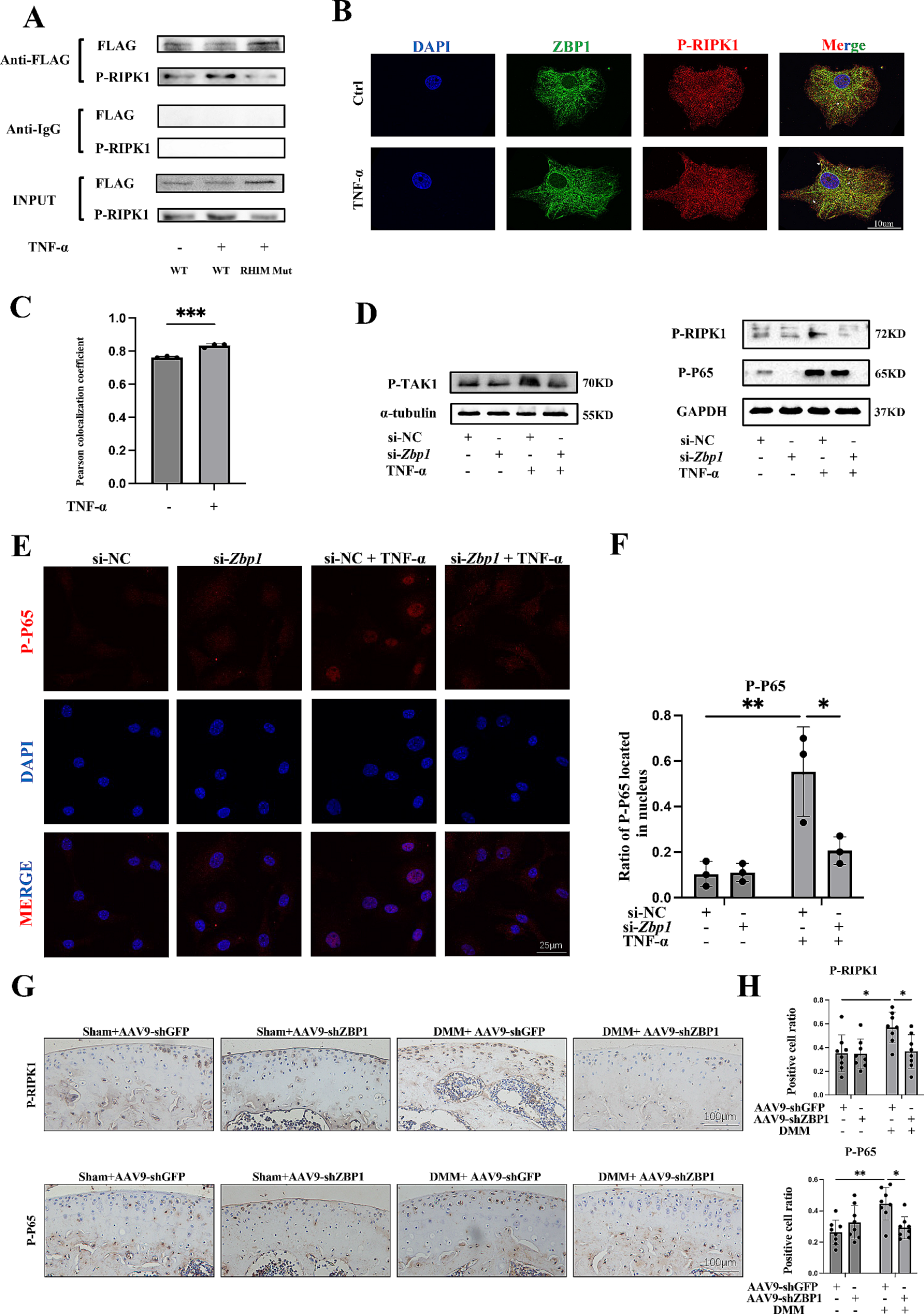
ZBP1 interacts with RIPK1 and activates the TAK1-NF-κB pathway. (A) Chondrocytes were infected by Adenoviruses expressing ZBP1 with FLAG (WT), a RHIM domain mutant ZBP1 with FLAG (MUT) following treatment with TNF-α for 15 min. The co-IP analysis detecting the binding of ZBP1 to RIPK1 is shown. (B) ZBP1 and P-RIPK1 colocalization in chondrocytes was detected using IF staining (*n* = 3; scale bar: 10 μm) and Pearson colocalization coefficient was shown in (C). (D) Western blot analysis of P-TAK1 expression in chondrocytes transfected with siNC or ZBP1 siRNA following TNF-α induction for 12 h (*n* = 3). Western blot analysis of P-RIPK1, and P-P65 expression in chondrocytes transfected with siNC or ZBP1 siRNA following TNF-α induction for 15 min. (E, F) Images of immunofluorescence staining and statistical analysis of relative P-P65 expression and its distribution in chondrocytes transfected with siNC or ZBP1 siRNA following TNF-α induction for 15 min (*n* = 3, scale bar: 25 μm). (G-H) IHC staining showing P-RIPK1-positive cells and P-P65-positive cells in the cartilage of the four groups (*n* = 8; scale bar: 100 μm). The data are shown as the means ± SDs. *P < 0.05, **P < 0.01, *** *P* < 0.001

Therefore, we established adenoviral vectors where the wild type (WT) virus contained the total sequence of ZBP1 and the mutant type virus contained the total sequence of ZBP1 in which the sequence of the RHIM domain was changed. Then, we performed co-IP experiments and found that ZBP1 could bind to p-RIPK1 and that TNF-α treatment could increase binding; mutation in the RHIM region caused this binding to be significantly reduced (Fig. 4A). Consistently, the IF colocalization results revealed increased colocalization of ZBP1 with P-RIPK1 in TNF-α-treated chondrocytes (Fig. 4B, C). The activation of RIPK1 can trigger the TAK1-NF-κB signaling pathway and mediate downstream cellular processes [36, 37]. Therefore, we investigated the impact of RIPK1 on this pathway, and the results showed that ZBP1 knockdown reduced the phosphorylation of TAK1, P65, and RIPK1 compared with that in the TNF-α group (Fig. 4D). The IF results also confirmed that knocking down ZBP1 reduced the nuclear translocation of P65 (Fig. 4E, F). In addition, we observed the effect of ZBP1 on IL-1β-treated chondrocytes and found that knocking down ZBP1 also repressed the phosphorylation of TAK1, P65, and the MAPK signaling pathway (Additional file 1: Fig. S4A, B). We further examined this phenomenon in vivo, and the IHC results revealed that AAV9-ZBP1 also reduced the expression levels of P-P65 and P-RIPK1 in articular cartilage compared to that of DMM group (Fig. 4G, H). Collectively, our data support the hypothesis that ZBP1 can interact with RIPK1 to activate the RIPK1-TAK1-NF-κB signaling pathway, which mediates chondrocyte inflammation and ECM degradation.

### ZBP1 expression depends on IRF1

**Fig. 5:**
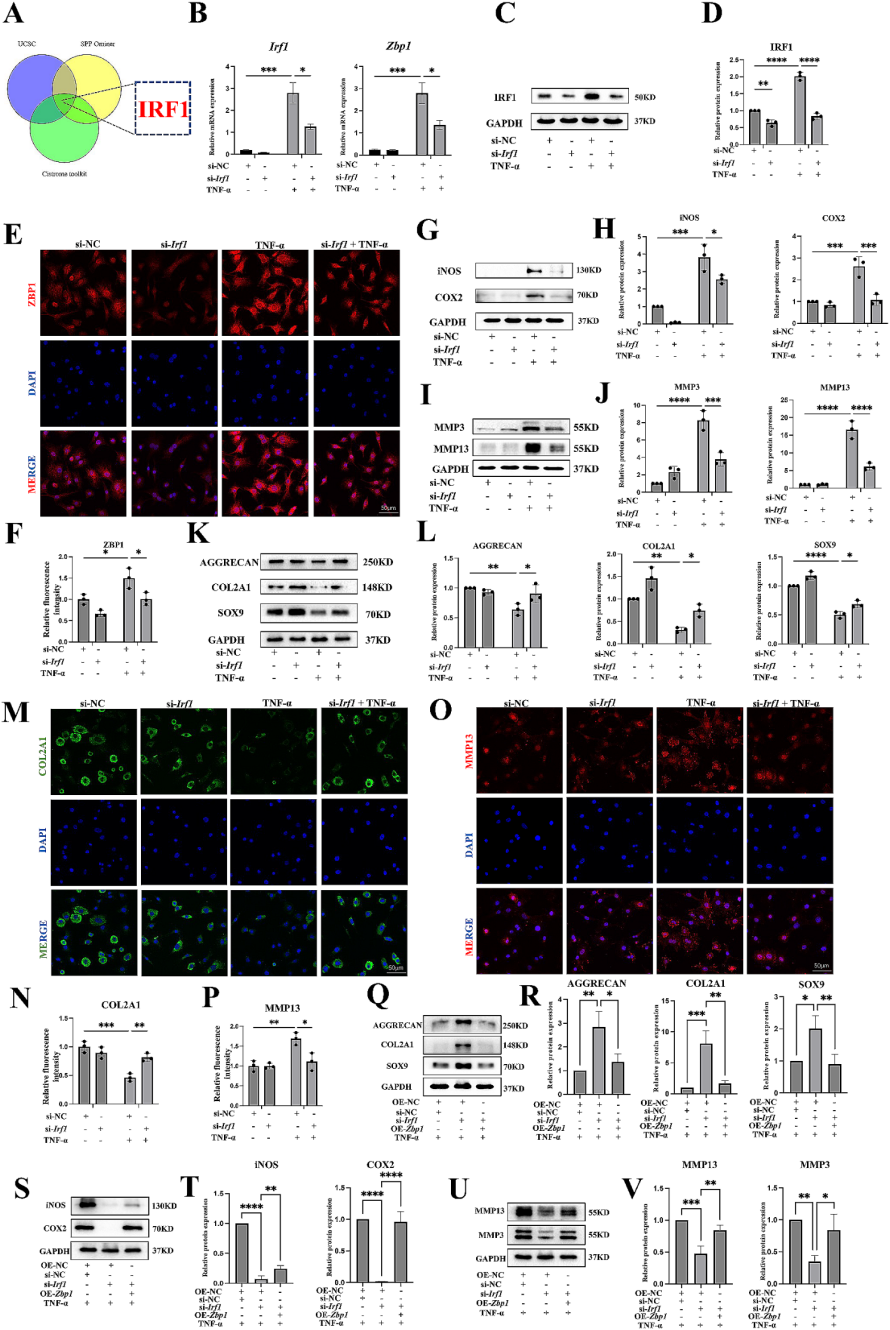
ZBP1 overexpression depends on IRF1. Chondrocytes were transfected with siNC or IRF1 siRNA for 24 h following TNF-α induction for 12 h. (A) Prediction of transcription factors using the UCSC Genome Browser database, SPP-ominer database, and Cistrome Data Browser. (B) qPCR results showing the relative mRNA expression levels of *Irf1* and *Zbp1* (*n* = 3). (C, D) Western blots and quantitative analysis of the relative protein expression level of IRF1 (*n* = 3). (E, F) Immunofluorescence staining and fluorescence intensity analysis of ZBP1 expression in chondrocytes transfected with siNC or IRF1 siRNA following TNF-α induction for 12 h (*n* = 3; scale bar: 50 μm). (G-L) Western blots and quantitative analysis of the relative protein expression levels of iNOS, COX2, MMP3, MMP13, AGGRECAN, COL2A1, and SOX9 (*n* = 3). (M-P) Immunofluorescence staining and fluorescence intensity analysis of COL2A1 and MMP13 expression in chondrocytes transfected with siNC or IRF1 siRNA following TNF-α induction for 12 h (*n* = 3; scale bar: 50 μm). Chondrocytes were transfected with negative control siRNA(siNC) or IRF1 siRNA (si-*Irf1*) or ZBP1-plasmid (OE-*Zbp1*) or empty plasmid (OE-NC) for 24 h following TNF-α induction for 24 h. (Q-V) Western blots and quantitative analysis of the relative protein expression of AGGRECAN, COL2A1, SOX9, iNOS, COX2 MMP13, and MMP3 in chondrocytes (*n* = 3). The data are shown as the means ± SDs. *P < 0.05, **P < 0.01, *** *P* < 0.001, **** *P* < 0.0001

The next key question is why ZBP1 expression is altered during OA development. The above results showed that the *Zbp1* mRNA level was increased by TNF-α treatment (Fig. 1I). This result suggested that ZBP1 expression may be affected by transcription. As a method to address this issue, we first used the UCSC Genome Browser database, Signaling Pathways Project (SPP)-ominer database and Cistrome Data Browser to predict the transcription factors regulating ZBP1 expression. Given that ZBP1 is an interferon-induced cytosolic nucleic acid sensor and IRF1 is a pivotal transcription factor of the interferon system [38, 39]. IRF1 was screened out as a possible transcription factor among the intersection of three database (Fig. 5A). Furthermore, we detected the *Irf1* and *Zbp1* mRNA expression levels under inflammatory conditions. qPCR results revealed that the *Irf1* expression level increased significantly at 1 h; however, the increase in *Zbp1* expression began at 6 h (Additional file 1: Fig. S5A). This time interval provided some support for our hypothesis. Then, an siRNA was used to knock down the expression of IRF1 in primary chondrocytes, and the knockdown efficiency was confirmed by qPCR (Fig. 5B and Additional file 1: Fig. S5B). Notably, the loss of IRF1 significantly attenuated both the mRNA and protein expression levels of ZBP1 (Fig. 5B-F). Moreover, IRF1 knockdown significantly attenuated TNF-α-induced chondrocyte damage, as revealed by a reduced inflammatory response, reduced ECM catabolism, and restored ECM anabolism (Fig. 5G-P). Functional rescue experiments were conducted to further prove that the expression of ZBP1 was regulated by IRF1. Interestingly, overexpression of ZBP1 reversed the protective effect of IRF1 knockdown on chondrocytes (Fig. 5Q-V). Overall, ZBP1 overexpression depends on the regulation of IRF1.

**CsA treatment inhibits the ability of ZBP1 to alleviate chondrocyte damage.**

**Fig. 6:**
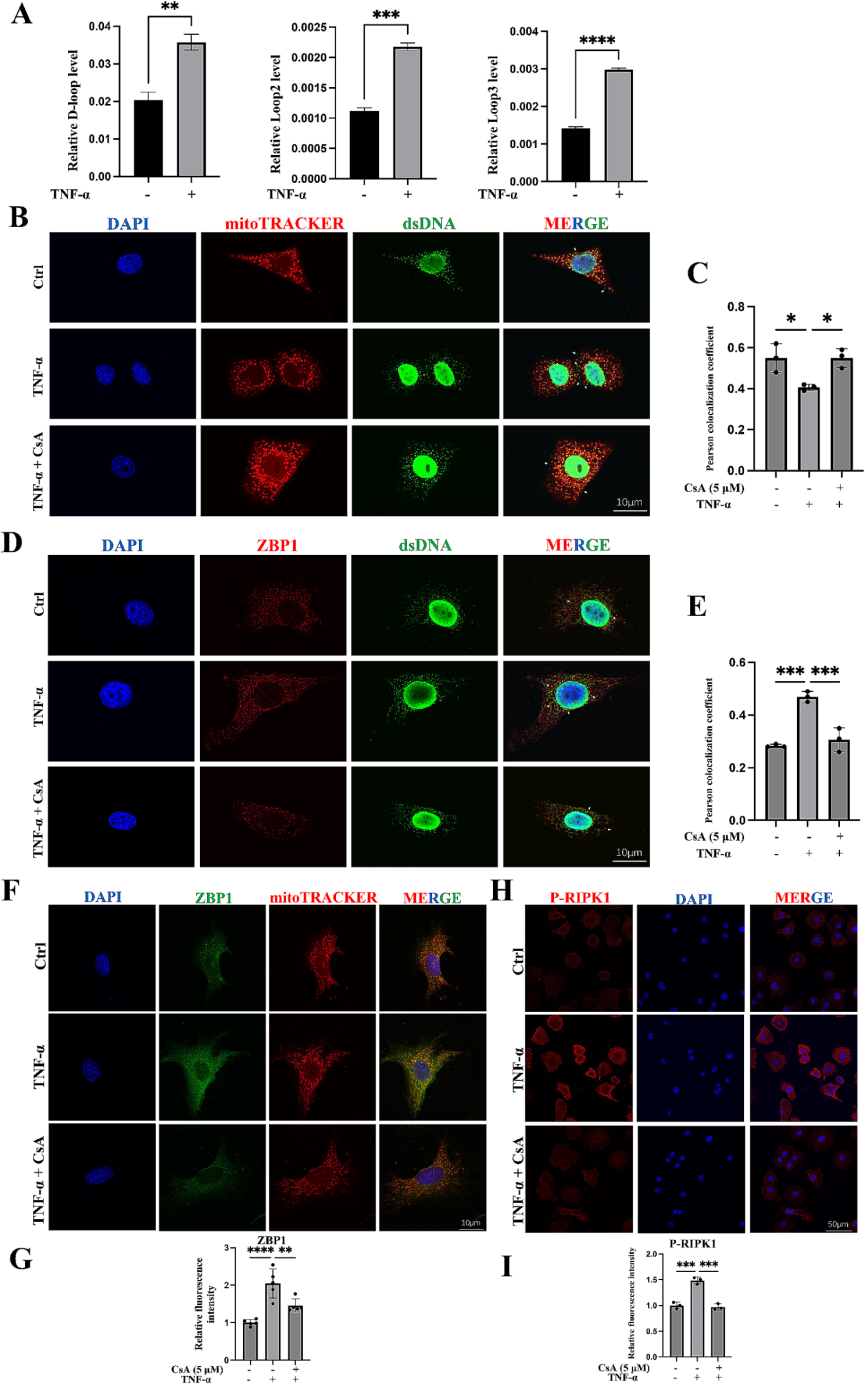
The effect of mtDNA on ZBP1. (A) qPCR results showing the relative mtDNA level in the cytoplasm compared to the total DNA level in chondrocytes treated with TNF-α for 12 h (*n* = 3; mtDNA: *D-loop*, *Loop2* and *Loop3*). Chondrocytes were treated with TNF-α alone or TNF-α combined with CsA for 12 h. (B) Images of immunofluorescence staining are shown; red indicates mitochondria, green indicates dsDNA, and white arrows indicate dsDNA released from mitochondria (*n* = 3; scale bar: 10 μm) and Pearson colocalization coefficient was shown in (C). (D) The colocalization of ZBP1 and dsDNA is shown (*n* = 3; scale bar: 10 μm) and Pearson colocalization coefficient was shown in (E). (F, G) Chondrocytes were treated with TNF-α alone or TNF-α combined with CsA for 48 h, immunofluorescence staining of ZBP1 and fluorescence intensity analysis. (H, I) Chondrocytes were treated with TNF-α alone or TNF-α combined with CsA for 15 min, immunofluorescence staining of P-RIPK1 and fluorescence intensity analysis. Data are shown as the means ± SDs. *P < 0.05, **P < 0.01, *** *P* < 0.001, **** *P* < 0.0001

Under conditions of cellular stress and mitochondrial dysfunction, mtDNA can be released into the cytoplasm or extracellular space to engage pattern recognition receptors, including ZBP1 [18]. Therefore, we further explored whether mtDNA released from OA chondrocytes regulates ZBP1. First, we extracted the mtDNA and total cellular DNA from the control and TNF-α groups. qPCR was used to assess the level of mtDNA, and we found that TNF-α led to mtDNA release into the cytoplasm compared with the control group, as evidenced by elevated levels of *D-loop*, *Loop2*, and *Loop3* (Fig. 6A). The opening of mitochondrial permeability transition pores (mPTPs) can enable the cytosolic release of mtDNA, and CsA has been verified to inhibit mPTP opening [17]. It was found that found that TNF-α caused more dsDNA released from mitochondria and more colocalization between ZBP1 and dsDNA, while CsA treatment not only decreased the mtDNA level in the cytoplasm (Fig. 6B, C) but also reduced the binding between ZBP1 and dsDNA in the cytoplasm compared to the TNF-α group (Fig. 6D, E). In addition, we also found that CsA reduced the TNF-α-induced expression levels of ZBP1 and p-RIPK1 (Fig. 6F-I). These data indicated that CsA could inhibited mtDNA-ZBP1-RIPK1 axis.

**Fig. 7:**
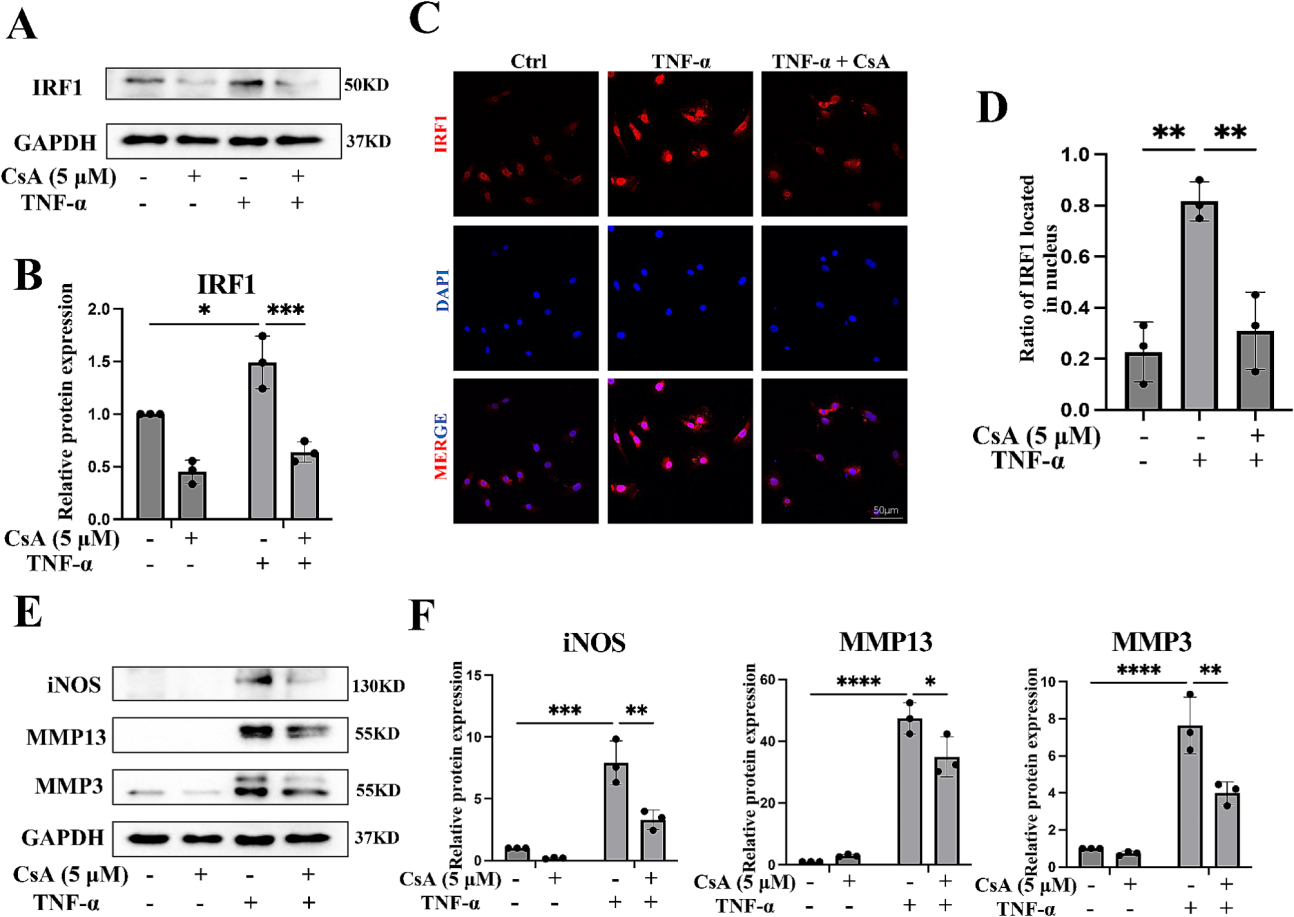
The effect of mtDNA on IRF1 and chondrocyte damage. Chondrocytes were treated with TNF-α alone or CsA alone or TNF-α combined with CsA for 12 h. (A, B) Western blots and quantitative analysis of expression level of IRF1 (*n* = 3). (C, D) immunofluorescence staining and fluorescence intensity analysis of IRF1 nuclear location (*n* = 3). (E, F) Western blots and quantitative analysis of expression level of iNOS, MMP13, and MMP3 (*n* = 3). Data are shown as the means ± SDs. *P < 0.05, **P < 0.01, *** *P* < 0.001, **** *P* < 0.0001

The previous study has indicated that IRFs could be regulated by mtDNA [40]. This finding prompts us to investigate the relationship between mtDNA and activation and expression of IRF1 and reduced the proportion of nuclear IRF1, which indicated reduced activation of IRF1, in TNF-α-treated chondrocytes (Fig. 7A-D). Furthermore, it was observed that the inflammatory response and ECM degradation in chondrocytes were significantly alleviated by CsA treatment (Fig. 7E, F). Collectively, these data indicated that mtDNA might play a dual role in the activity and expression of ZBP1 to cause chondrocyte damage, suggesting that the inhibition of mtDNA release with CsA protects against TNF-α-treated chondrocyte damage.

### The administration of CsA attenuates murine OA progression

**Fig. 8:**
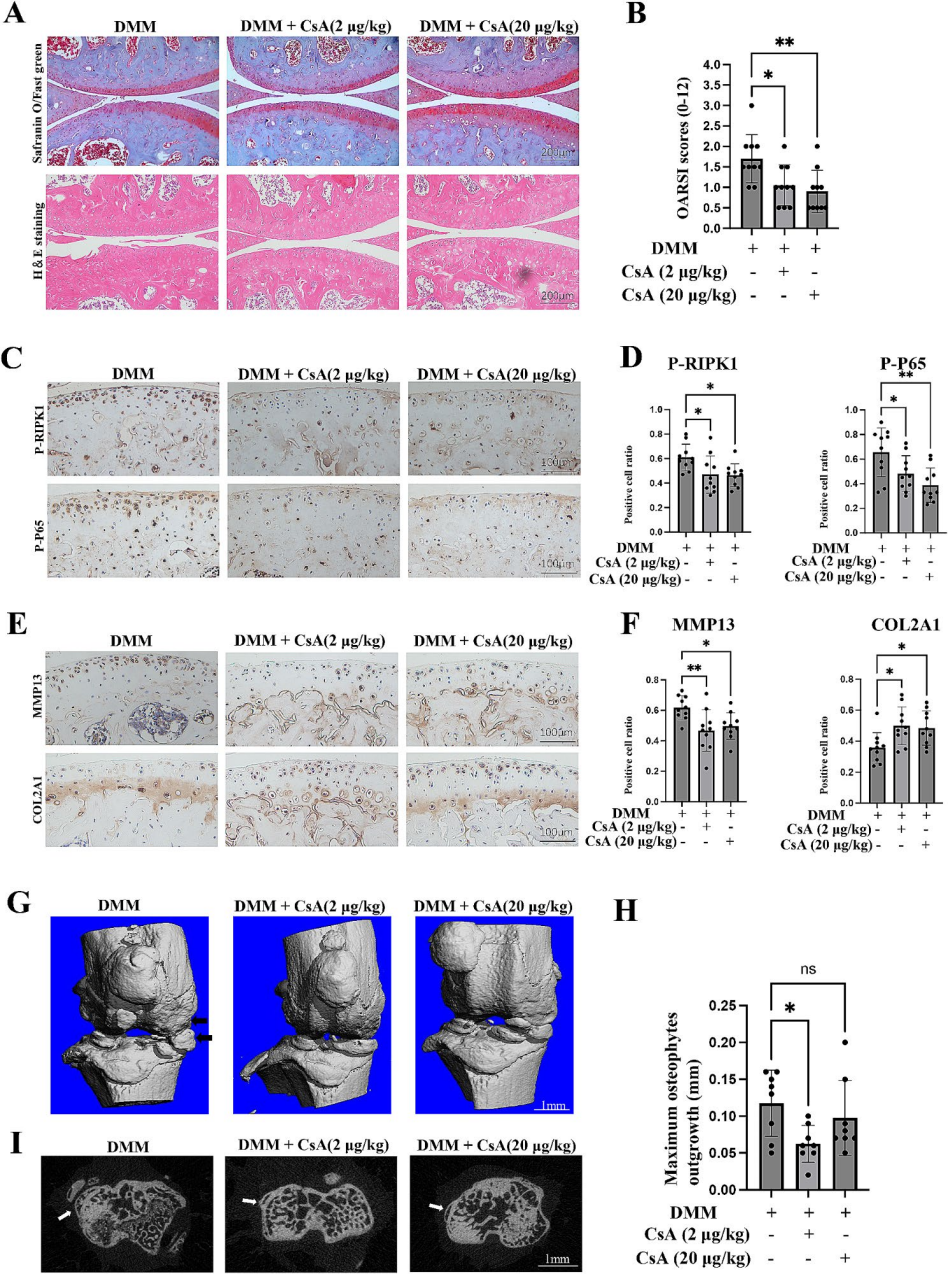
Inhibition of the mtDNA-IRF1-ZBP1 axis with CsA protects mice from DMM-induced OA progression. (A, B) Safranin O/fast green staining, HE staining and OARSI scores of the three groups (DMM, *n* = 10; DMM + CsA (2 µg/kg), *n* = 10; DMM + CsA (20 µg/kg), *n* = 10) (scale bar: 200 μm). (C, D) IHC staining showing p-P65-positive cells and p-RIPK1-positive cells in the cartilage of the three groups (scale bar: 100 μm). (E, F) IHC staining showing COL2A1-positive cells and MMP13-positive cells in the cartilage of the three groups (scale bar: 100 μm). (G) Images of 3D reconstructions of mouse joints; the black arrow indicates the osteophyte (scale bar: 1 mm). (H, I) Transverse sectional images and statistical analysis of the maximum osteophyte outgrowth of each joint in the three groups; the white arrow indicates the osteophyte (DMM, *n* = 8; DMM + CsA (2 µg/kg), *n* = 8; DMM + CsA (20 µg/kg), *n* = 8; scale bar: 1 mm). The data are shown as the means ± SDs. *P < 0.05, **P < 0.01, NS indicates not significant

Based on the above findings, we sought to validate whether inhibiting the mtDNA-IRF1-ZBP1-RIPK1 axis with CsA could protect against OA progression. Different doses of CsA (2 µg/kg and 20 µg/kg) were intra-articularly injected into the joint cavity to achieve local administration. One week after DMM surgery, CsA or vehicle was injected into the joint cavity once a week for 7 continuous weeks. Importantly, CsA administration significantly attenuated cartilage degeneration, as illustrated by Safranin O/fast green staining, HE staining, and a decreased OARSI score (Fig. 8A, B). Consistently, CsA-treated mice manifested lower levels of MMP13, P-RIPK1, and P-P65, together with higher levels of COL2A1 in cartilage than DMM mice (Fig. 8C-F). Moreover, osteophyte formation and subchondral bone formation in the three groups were compared, and the results indicated that osteophyte formation was significantly reduced by CsA treatment, as indicated by a lower osteophyte score and shorter osteophyte outgrowth in the CsA group than in the DMM group (Fig. 8G, H). However, CsA did not significantly change the subchondral bone mass (Additional file 1: Fig. S6A). Taken together, our data indicated that CsA markedly reduced cartilage degeneration and osteophyte formation in OA mice.

## Discussion

ZBP1 is related to innate immunity and a variety of inflammatory diseases [41], but the link between ZBP1 and OA is fully unknown. To our knowledge, this report is the first showing that ZBP1 can regulate inflammatory chondrocytes and contribute to murine OA progression. In our previous study, we identified an essential role for RIPK1 in chondrocyte damage and OA progression, suggesting that the regulation of RIPK1 has an important impact on OA [34, 35, 42]. Furthermore, in this study, we reported that ZBP1 interacted with RIPK1 and elicited downstream cascades, including the activation of NF-κB signaling and ECM degradation. This finding is consistent with the findings that ZBP1 promotes the LPS-induced inflammatory response in macrophages via RHIM-mediated interactions with RIPK1 [43]. These data provide a theoretical basis for the involvement of ZBP1-RIPK1 in the development of OA.

Considering the role of ZBP1 in inflammation-associated diseases, elucidating the regulatory mechanism of ZBP1 expression and activity is important to more precisely target this molecule. Previous studies have suggested that ZBP1 expression can be regulated by interferons [12] or heat shock transcription factor 1 under heat stress conditions [44]. Unfortunately, these conditions seem to have little impact on OA pathogenesis. This gap prompted us to identify a novel regulator of ZBP1. The most exciting discovery in this study is that ZBP1 overexpression occurred in an IRF1-dependent manner, and the loss of IRF1 led to protective effects similar to those of ZBP1 knockdown on chondrocytes.

Released mtDNA acts as a pathological molecule and activator of ZBP1 in various diseases [18, 45, 46]. Although the synovial fluid mtDNA concentration reflects the degree of cartilage damage [19], how mtDNA mediates chondrocyte damage has yet to be determined. In this context, we proposed that inflammatory factors may cause mitochondrial damage and release mtDNA into the cytoplasm to participate in ZBP1 activation. Indeed, we found that TNF-α could cause mtDNA release and binding to ZBP1, while the inhibition of mtDNA release with CsA reversed the above phenomenon and the OA-like phenotype of chondrocytes. Interestingly, we also observed that IRF1 expression was downregulated by CsA treatment, indicating that mtDNA might exert dual regulatory effects on ZBP1 expression and activity. Collectively, these data provide evidence that the IRF1-ZBP1 axis links mtDNA and chondrocyte damage during OA development.

After identifying mtDNA-IRF1-ZBP1-RIPK1 as the pathological axis involved in OA pathogenesis, we sought to identify a viable target against OA by inhibiting this axis. Given that mtDNA acts upstream of this axis and that mtDNA release could be effectively blocked by CsA, the therapeutic effect of CSA was assessed on OA mice. Importantly, intra-articular delivery of CsA protected mice from DMM-induced cartilage destruction and osteophyte formation. Additionally, CsA is an FDA-approved drug that is widely applied in clinical practice [47]. This information could aid in the development of CsA as a therapeutic agent for OA.

This study has several limitations. Firstly, although we clarified that ZBP1 was upregulated in an IRF1-dependent manner, the specific binding region involved has yet to be fully elucidated. Secondly, mtDNA can be released by several channels; however, we investigated only the mPTP and did not evaluate voltage-dependent anion-selective channels, which is a major focus of our follow-up studies. Thirdly, CsA is a powerful immunosuppressive drug and might cause side effects in the clinic [48]. The precise dose and therapeutic window of CsA should be optimized in preclinical research. CsA is not specific inhibitor of mtDNA release and CsA plays a role in multiple signaling pathways [48, 49]. We failed to exclude the other possible effects that CsA causes. In addition, the cartilage of eight-week-old mice is not totally mature, which preserves the heal capacity, properly causing a mild cartilage degeneration in OA model. the twelve-week-old mice and aging mice will be used as the model in the future studies.

## Conclusions

**Fig. 9:**
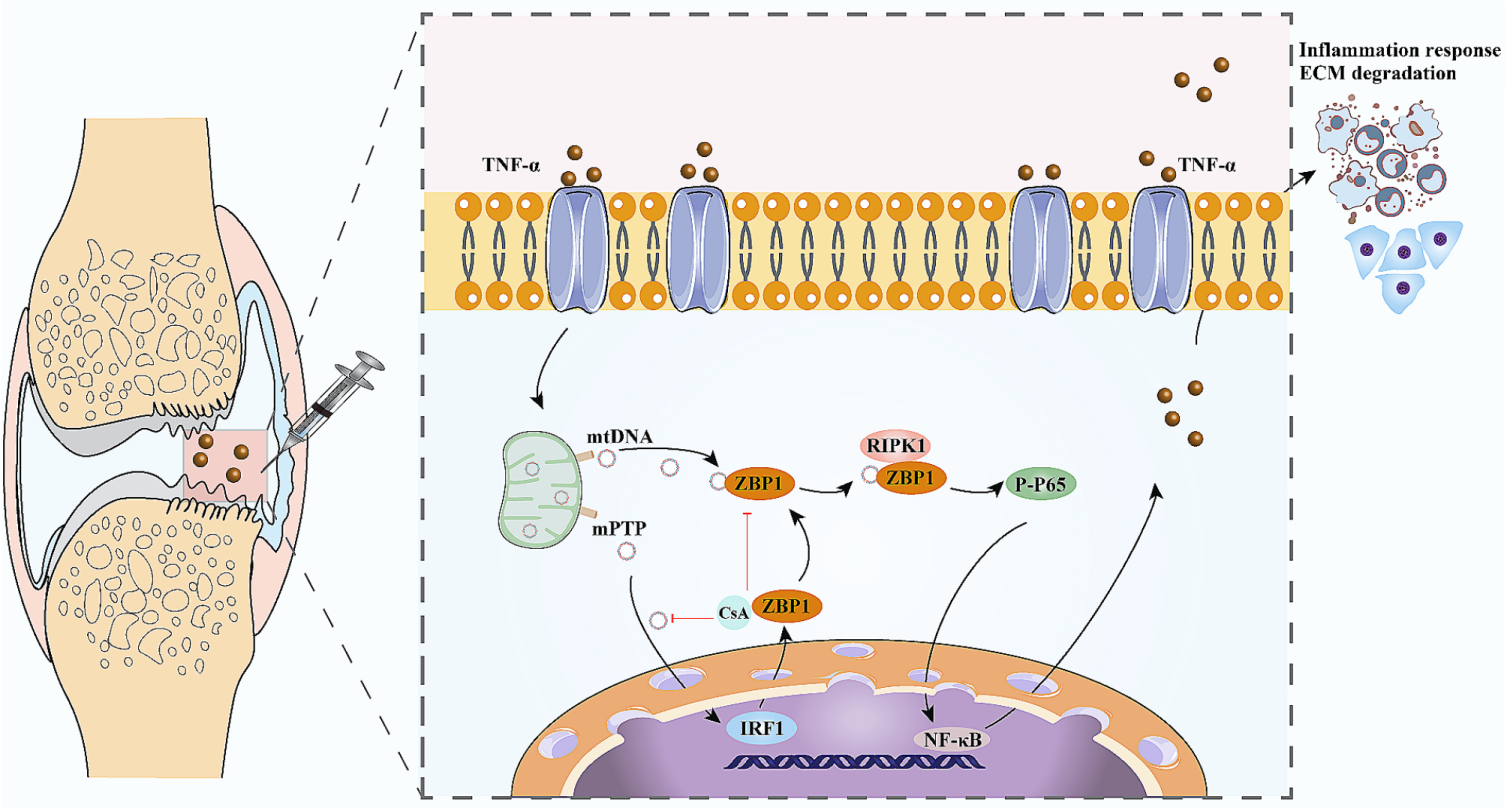
Graphical illustration of the role of the mtDNA-IRF1-ZBP1 axis in chondrocyte damage and OA pathogenesis. TNF-α is released into extracellular fluid in OA conditions. TNF-α could stimulate the chondrocytes and cause damage to the mitochondria, resulting in the release of mtDNA into the cytoplasm. The released mtDNA can not only bind to ZBP1 to activate ZBP1, but also stimulate IRF1 transcription which resulting in an increase in the expression of ZBP1. Later, activated ZBP1 can bind and interact with P-RIPK1, causing phosphorylation of TAK1 and P65, leading to the activation of NF-κB pathway which contributes to inflammatory response and ECM degradation. Inhibition of the mtDNA-IRF1-ZBP1 axis with CsA could alleviate chondrocyte damage and DMM-induced OA progression

In summary, this study revealed a positive correlation between ZBP1 expression and OA. We also identified mtDNA-IRF1-ZBP1-RIPK1 as the pathological axis involved in chondrocyte damage. In addition, inhibition of the mtDNA-IRF1-ZBP1 axis with CsA protected mice from DMM-induced OA progression (Fig. 9). Our findings highlight the essential role of ZBP1 in linking mtDNA and chondrocyte damage and reveal a new axis that may be used as a target for OA therapy.

### Electronic supplementary material

Below is the link to the electronic supplementary material.

Supplementary Material 1

Supplementary Material 2

## Data Availability

No datasets were generated or analysed during the current study.

## Abbreviations

AAV9: adeno-associated virus 9
BV/TV: bone volume/total volume
co-IP: coimmunoprecipitation
COL2A1: collagen type II α 1 chain
COX2: cyclooxygenase-2
CsA: cyclosporine A
DAMPs: danger-associated molecular patterns
DMM: destabilization of the medial meniscus
dsDNA: double-stranded DNA
ECM: extracellular matrix
HE: hematoxylin–eosin
IF: immunofluorescence
IHC: immunohistochemistry
IL-1β: interleukin-1β
IRF1: interferon regulatory factor 1
micro-CT: microcomputed tomography
mtDNA: mitochondrial DNA
MMP3: matrix metallopeptidase 3
MMP13: matrix metallopeptidase 13
mPTP: mitochondrial permeability transition pore
NC: negative control
OA: osteoarthritis
OARSI: Osteoarthritis Research Society International
PAMPs: pathogen-associated molecular patterns
RHIM: RIP homotypic interaction motif
siRNA: small interfering RNA
Tb.N: trabecular number
Tb.Sp: trabecular separation
Tb.Th: trabecular thickness
TNF-α: tumor necrosis factor-α
VDAC: Voltage-Dependent Anion Channel
ZBP1: Z-DNA binding protein 1
