# Supplementary material for: IRF1 regulation of ZBP1 links mitochondrial DNA and chondrocyte damage in osteoarthritis
Source: Cell Commun Signal. 2024 Jul 18;22:366. doi: 10.1186/s12964-024-01744-1 (PMC11256489; doi:10.1186/s12964-024-01744-1)
Supplement: Supplementary file 1 — Supplementary Material 1 [file 12964_2024_1744_MOESM1_ESM.docx]

**Table S1.** Amputees’ information

| Sample ID | Age | Gender | Height(cm) | Weight(kg) | amputation |
| --- | --- | --- | --- | --- | --- |
| 1 | 32 | Male | 175 | 79 | Right |
| 2 | 43 | Male | 168 | 65 | Right |
| 3 | 38 | Male | 173 | 71 | Left |
| 4 | 46 | Male | 171 | 68 | Right |
|  |  |  |  |  |  |

**Table S2.** OA patients’ information

| Sample ID | Age | Gender | Height(cm) | Weight(kg) | OA location |
| --- | --- | --- | --- | --- | --- |
| 1 | 67 | Male | 170 | 84 | Left |
| 2 | 66 | Female | 158 | 60 | Right |
| 3 | 72 | Male | 172 | 72 | Left |
| 4 | 67 | Female | 163 | 58 | Left |
| 5 | 61 | Female | 160 | 60 | Left |
| 6 | 74 | Male | 172 | 77 | Right |
| 7 | 75 | Female | 165 | 62 | Left |
| 8 | 72 | Male | 178 | 88 | Right |
| 9 | 78 | Male | 156 | 52 | Left |
| 10 | 69 | Female | 163 | 73 | Left |
| 11 | 78 | Female | 162 | 65 | Right |
| 12 | 76 | Female | 159 | 68 | Right |

**Figure S1** ****

**A**

**B**

**Fig. S1.** (A) The knockdown efficiency of ZBP1 using siRNA. Primary chondrocytes were transfected with scrambled siRNA or si-ZBP1 (sequence#1, sequence#2 and sequence#3). (B) qRT-PCR result of the overexpression-ZBP1 effciency. Data are shown as mean ± SD. *** P<0.001****, P<0.0001*.

**Figure S2**

**A**


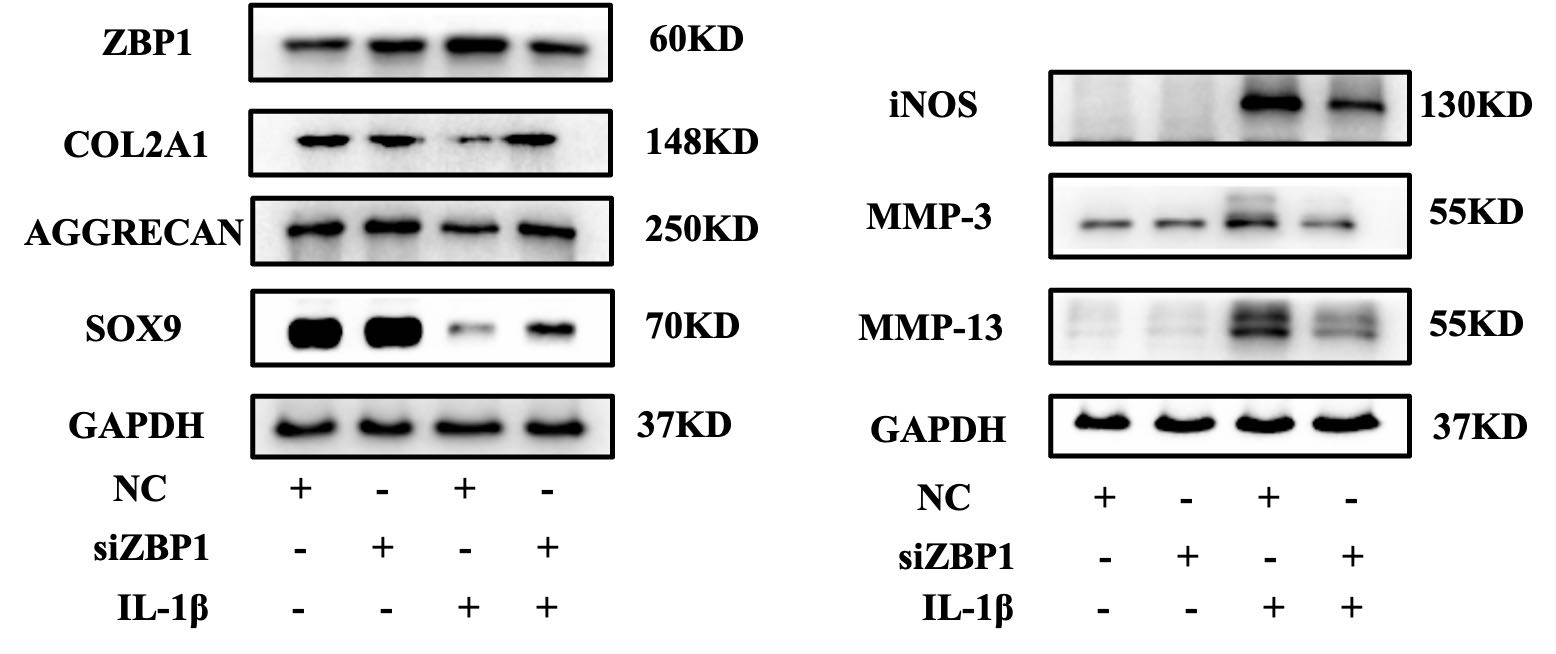


**** ****

**B**

**Fig. S2.** Chondrocytes were transfected with siNC or siZBP1 siRNA following IL-1β induction for 12 h. (A) Western blot to detect the expression of AGGRECAN, COL2A1, SOX9, iNOS, COX2, MMP3, and MMP13. (B) qPCR of *Mmp13*, *Cox2*, and *iNOS* relative expression level. Data are shown as mean ± SD. *P < 0.05, *** P < 0.001, **** P < 0.0001.

**Figure S3**

**A**

**Fig. S3**. (A) The analysis of BV/TV, Tb. N, Tb. Th and Tb. Sp between the four groups (Sham + AAV9-GFP, n = 8; Sham + AAV9-ZBP1, n = 8; DMM + AAV9-GFP, n = 8; DMM + AAV9-ZBP1, n = 8). Data are shown as mean ± SD. *P < 0.05, ** P < 0.01.

**Figure S4**

**B**

**A**

**Fig. S4.** (A, B) Treat the chondrocytes with IL-1β for 15 min and the western blot experiment was conducted to detect the relative expression level of P-TAK1, P-P65, P-ERK, P-P38, P-JNK.

**Figure S5**

**A**

***Irf1***

***Zbp1***


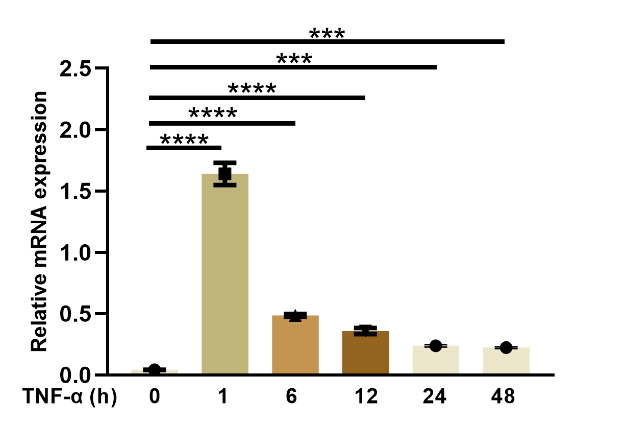

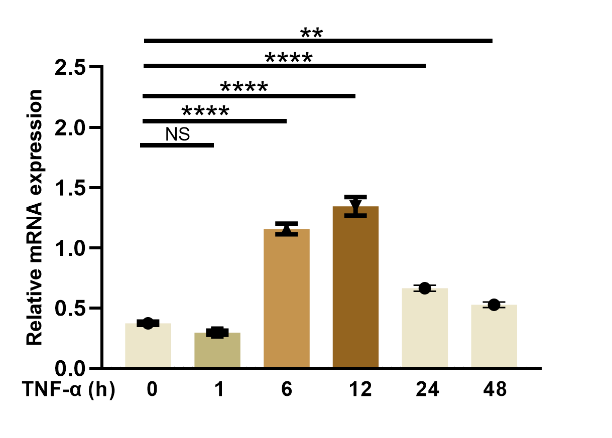

**B**

**Fig. S5.** (A) qPCR results of *Irf1* and *Zbp1* relative expression level in chondrocytes treated with TNF-α for 0, 1, 6, 12, 24 and 48 h. (B) The knockdown efficiency of IRF1 using siRNA. Primary chondrocytes were transfected with scrambled siRNA or si-IRF1 (sequence#1, sequence#2, and sequence#3).

**Figure S6**

**A**

**Fig. S6.** (A) the analysis of BV/TV, Tb. N, Tb. Th and Tb. Sp between the three groups (DMM, n = 10; DMM+CsA (5 μM), n = 10; DMM + CsA (50 μM), n = 10). Data are shown as mean ± SD. *P < 0.05, ** P < 0.01.
