# Supplementary figures and images for: IRF1 regulation of ZBP1 links mitochondrial DNA and chondrocyte damage in osteoarthritis
Source: Cell Commun Signal. 2024 Jul 18;22:366. doi: 10.1186/s12964-024-01744-1 (PMC11256489; doi:10.1186/s12964-024-01744-1)

Fig.1

F

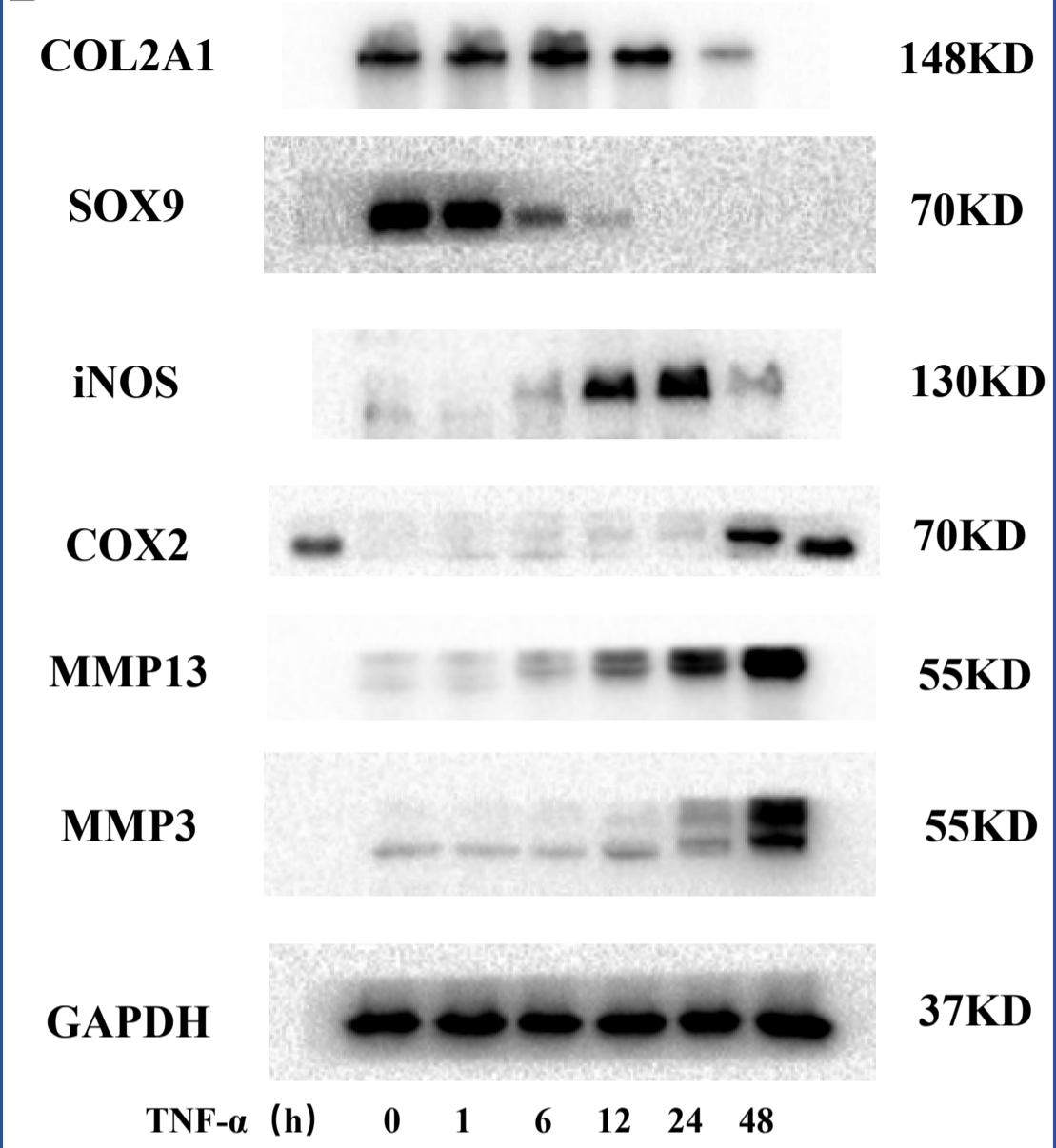

G

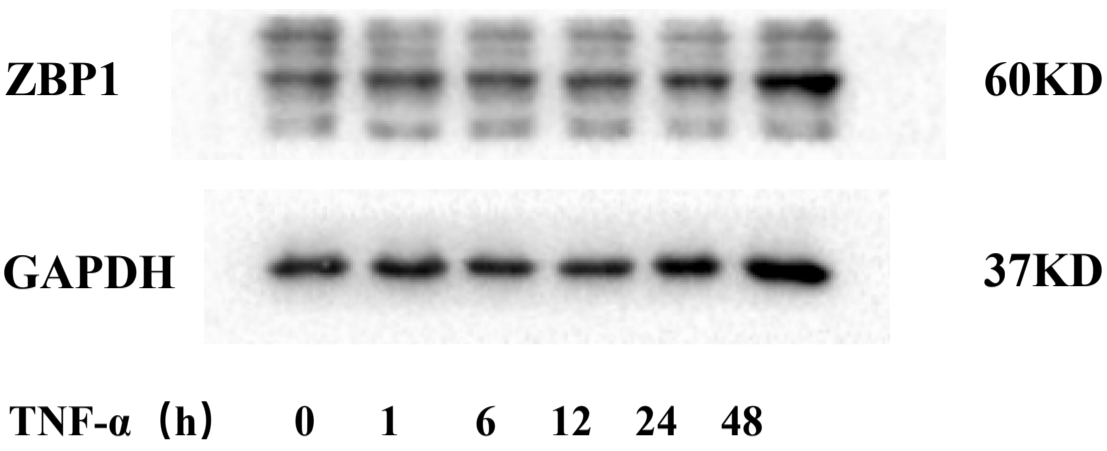

Fig.2

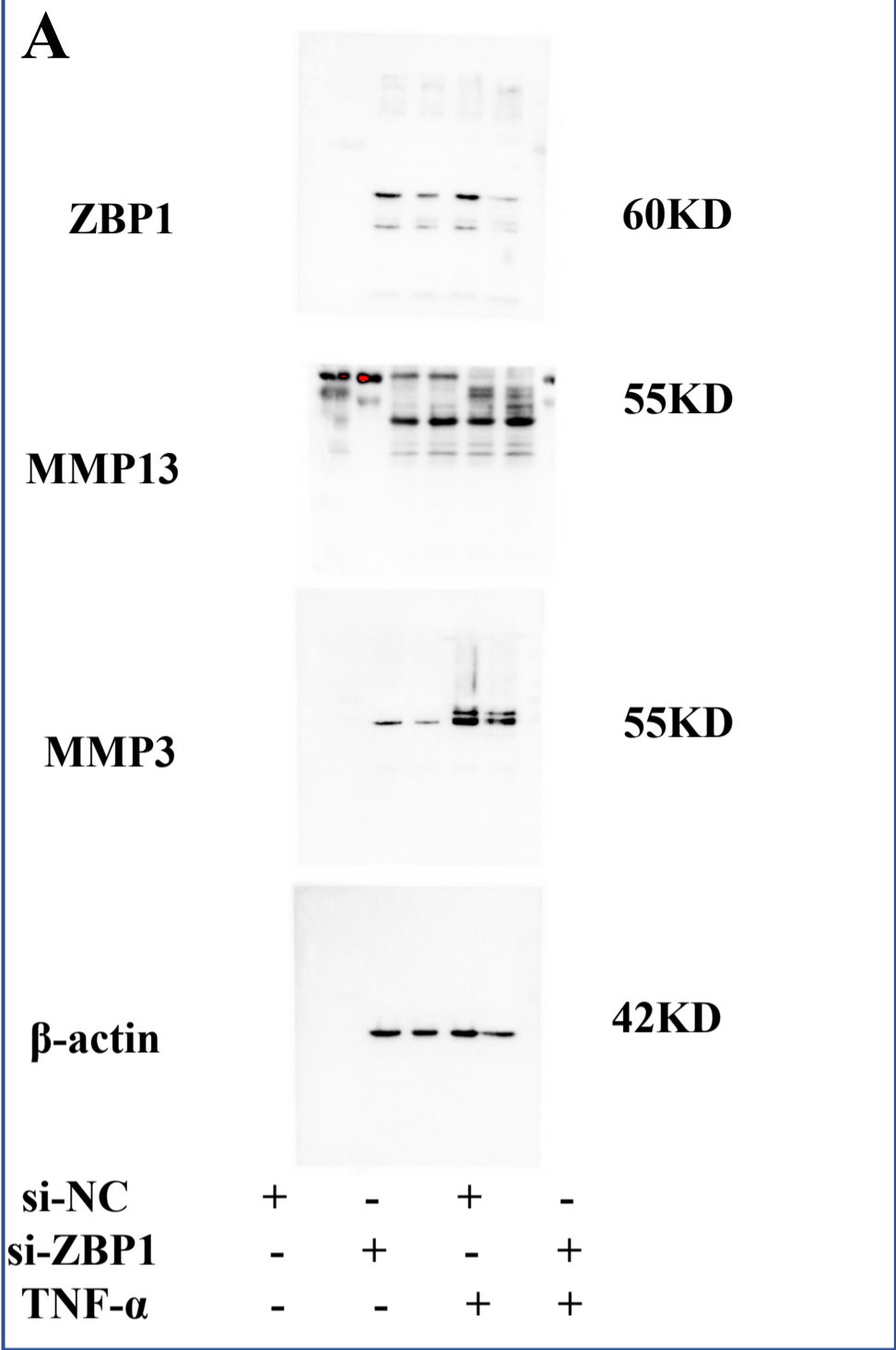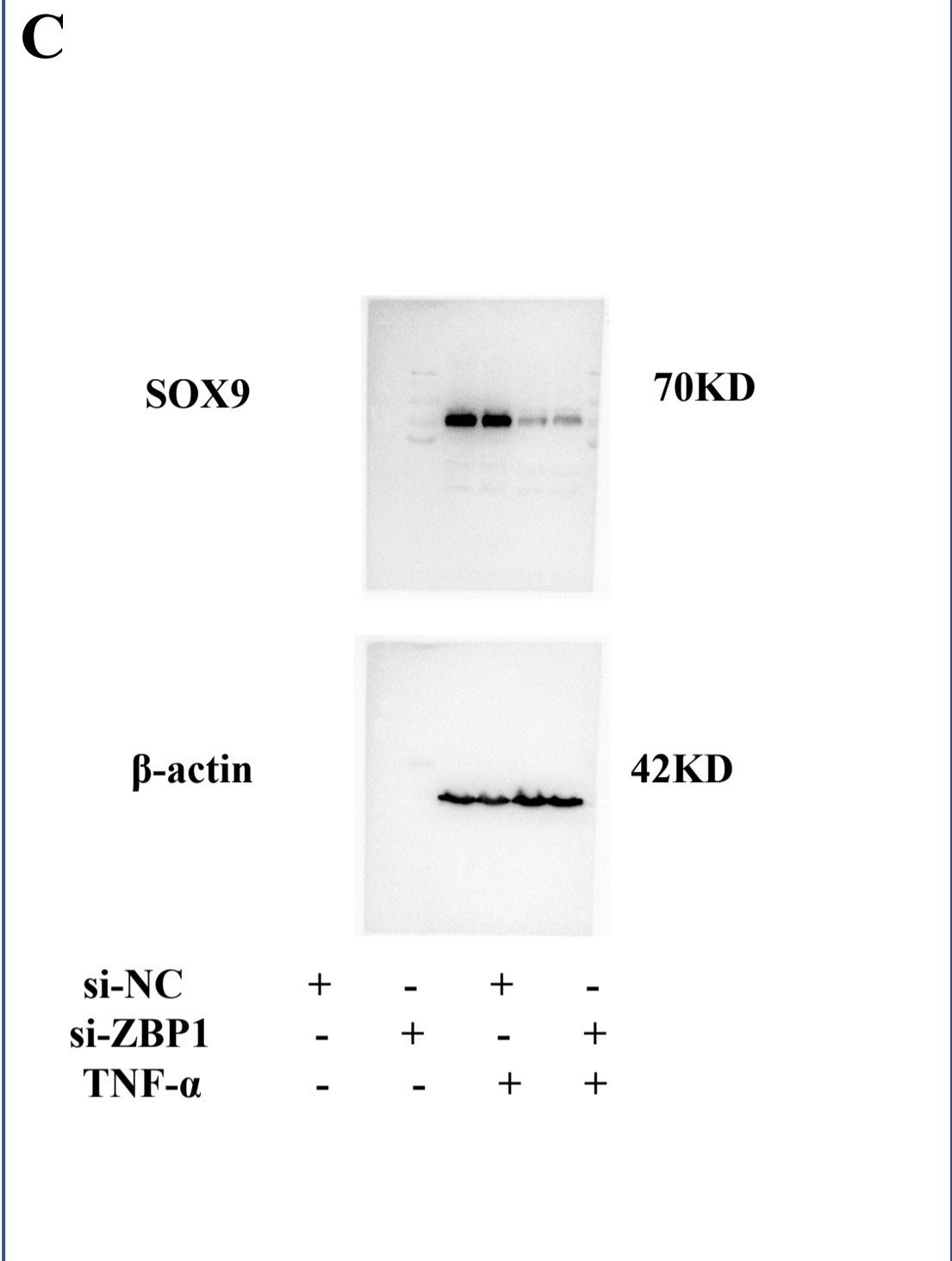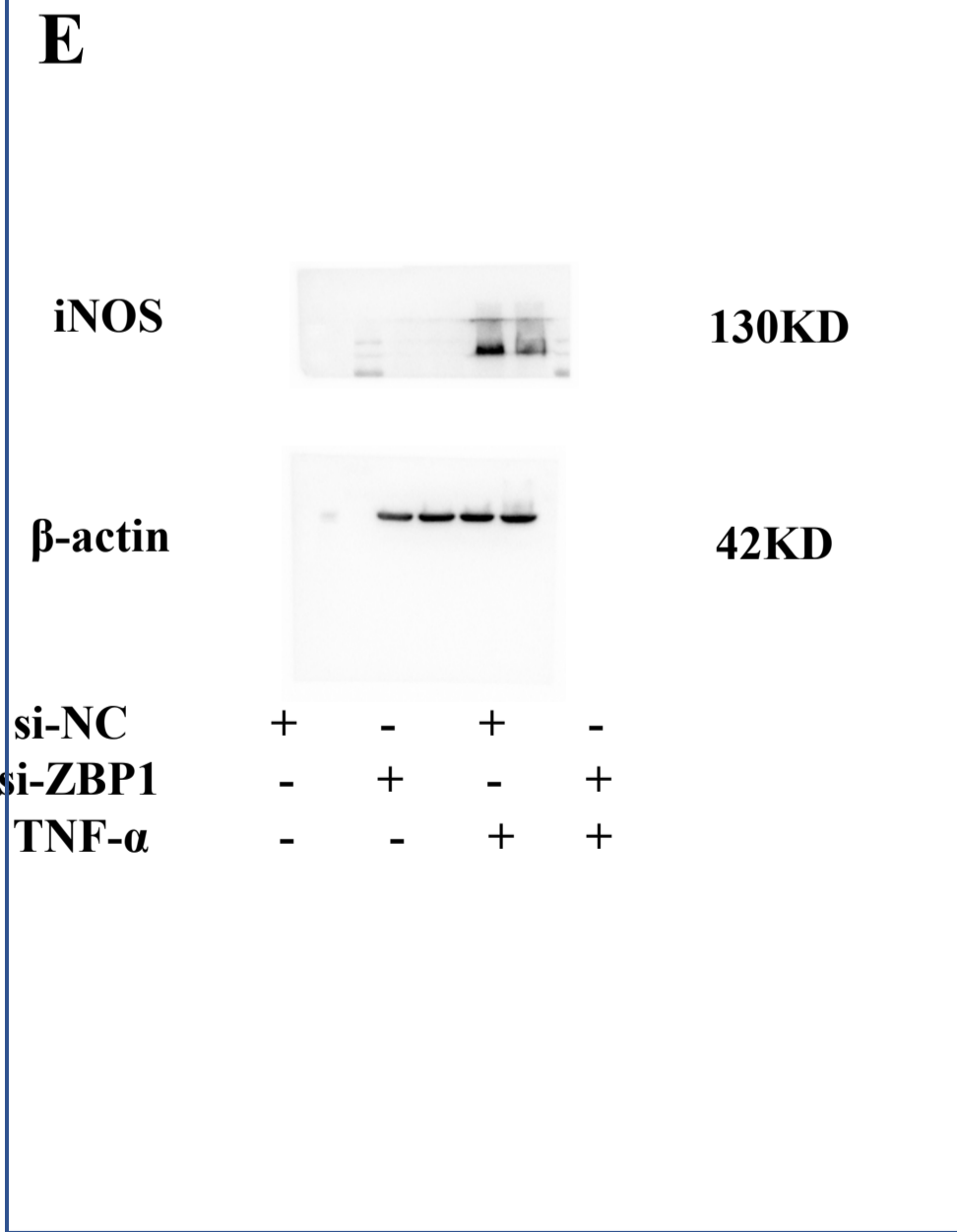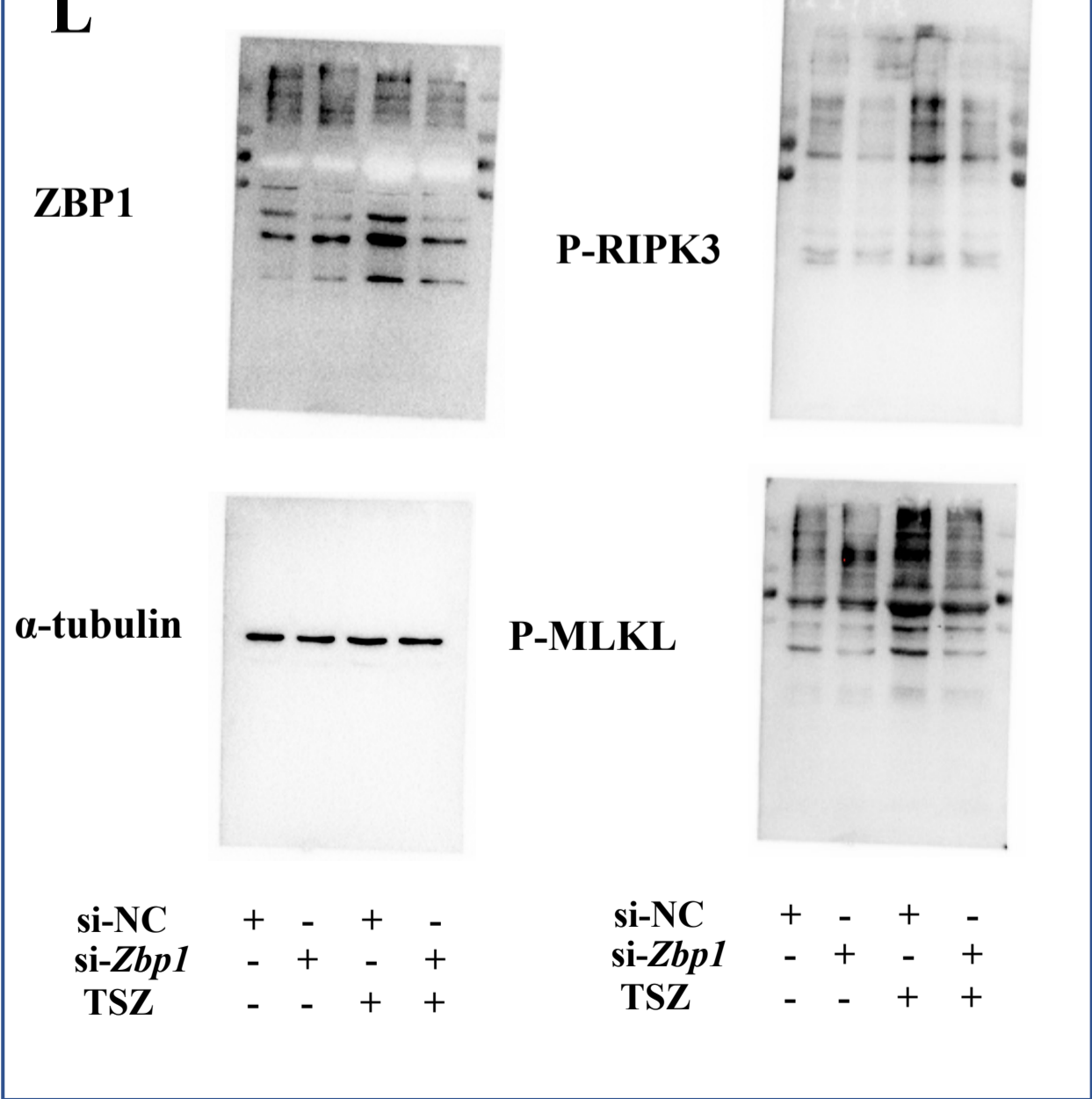

Fig.4

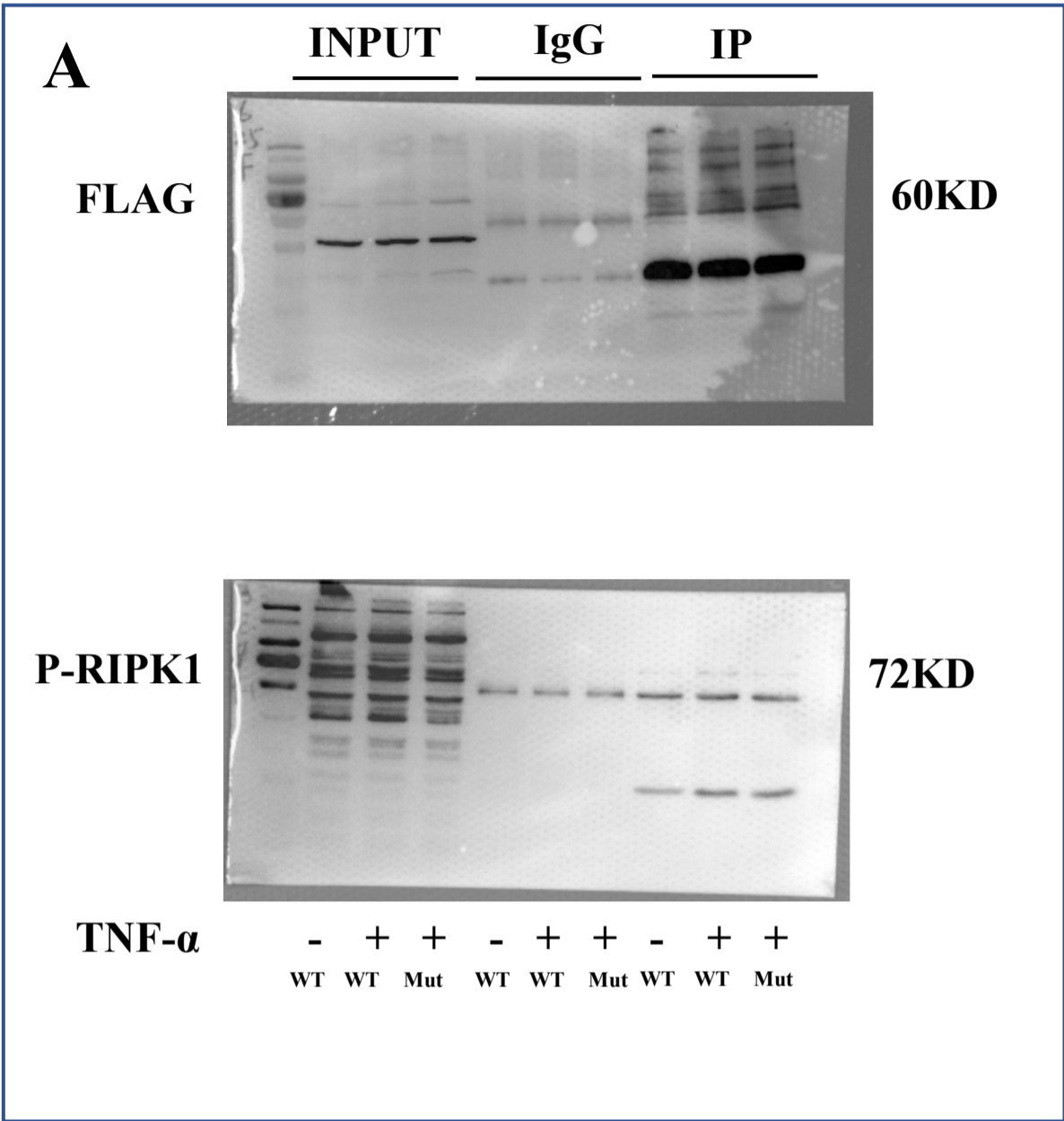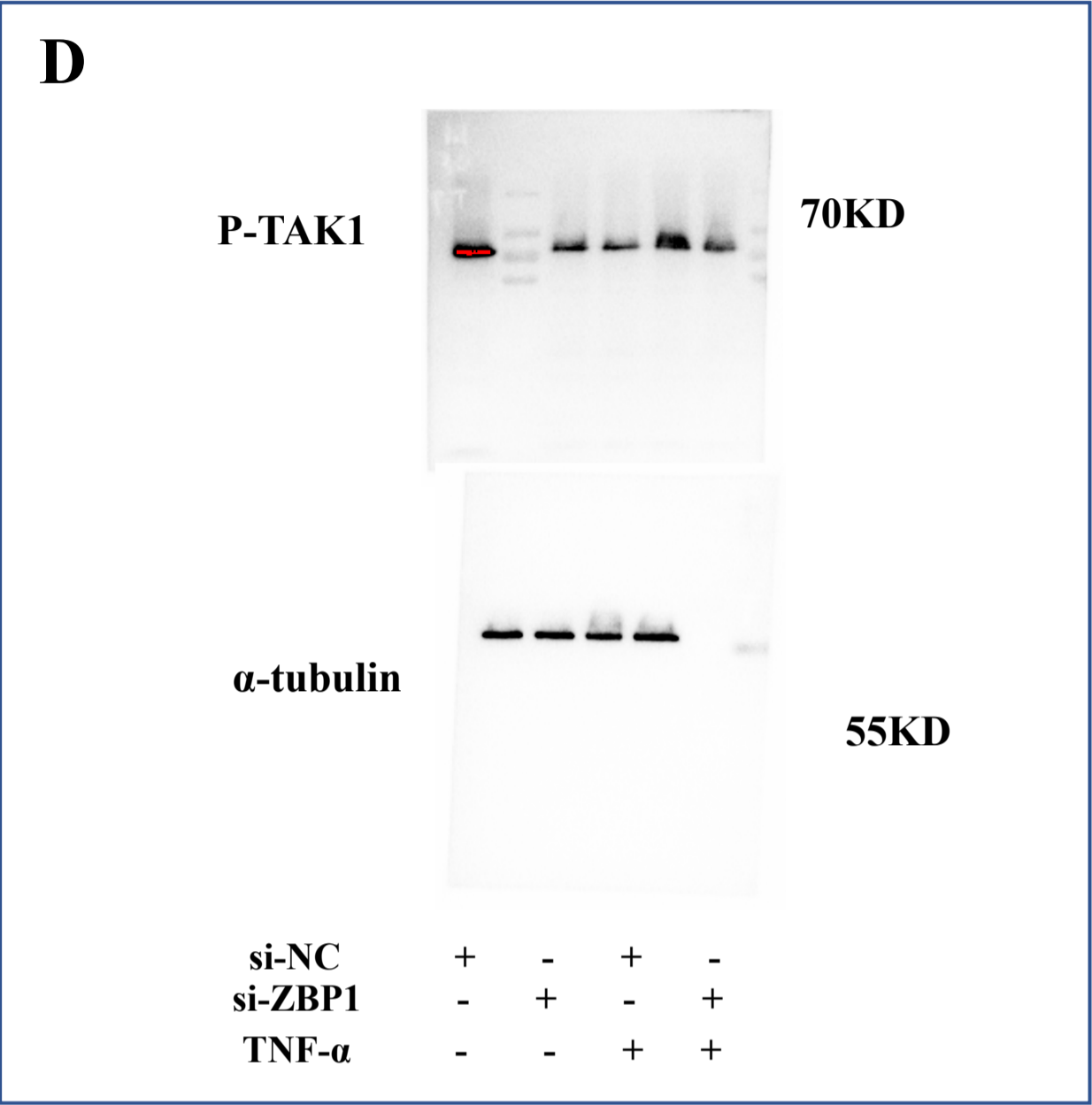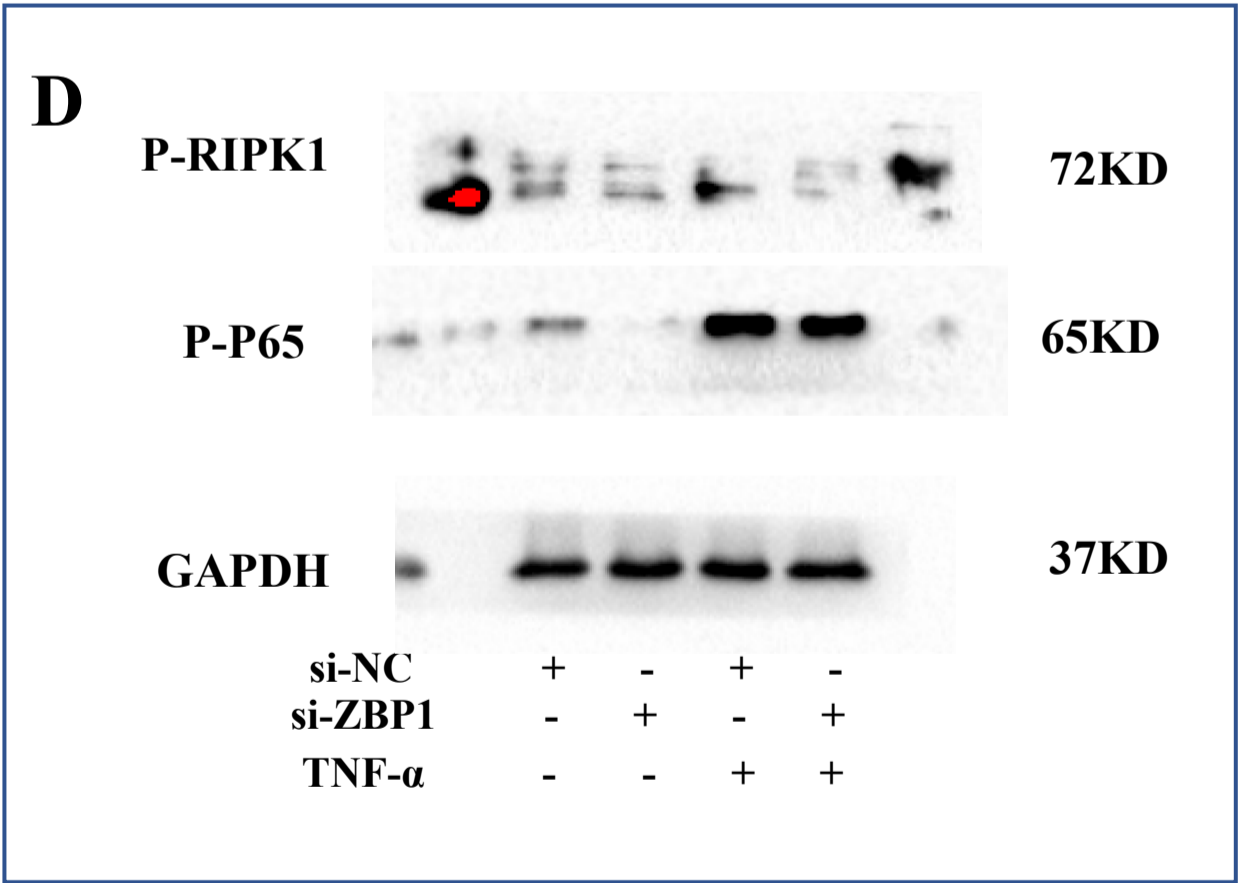

Fig.5

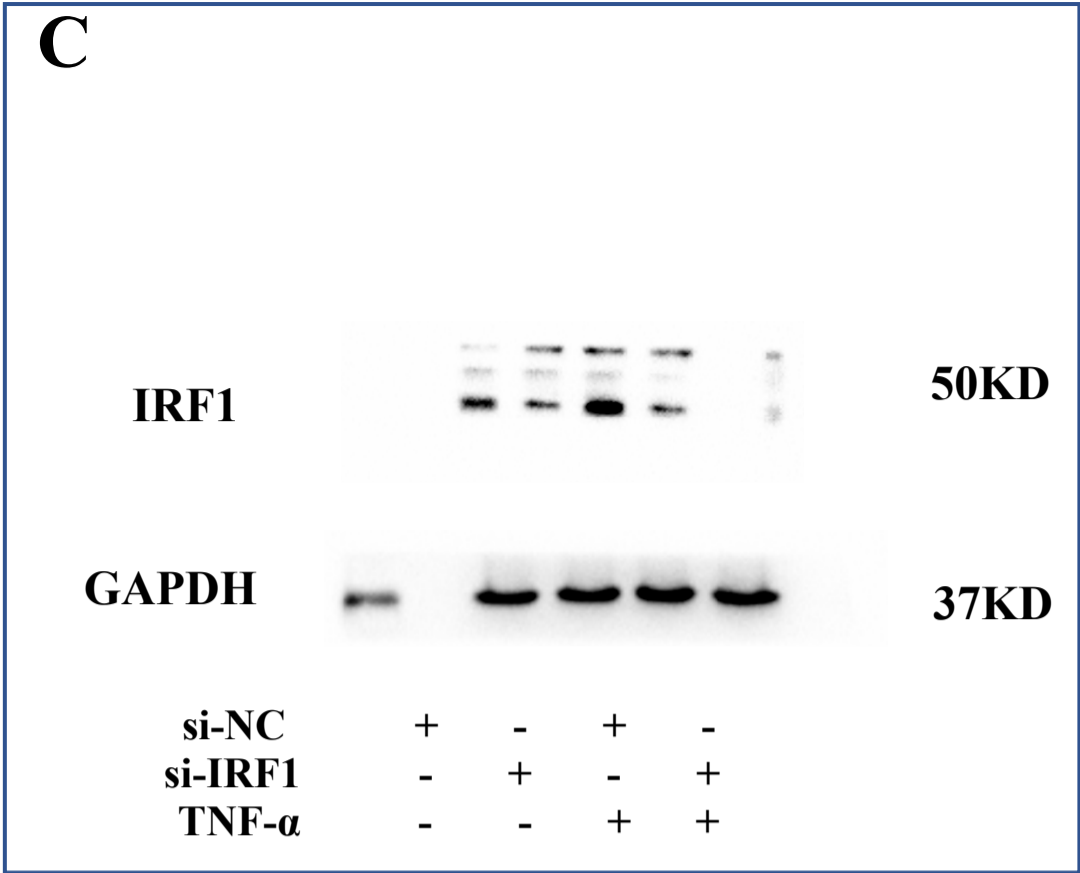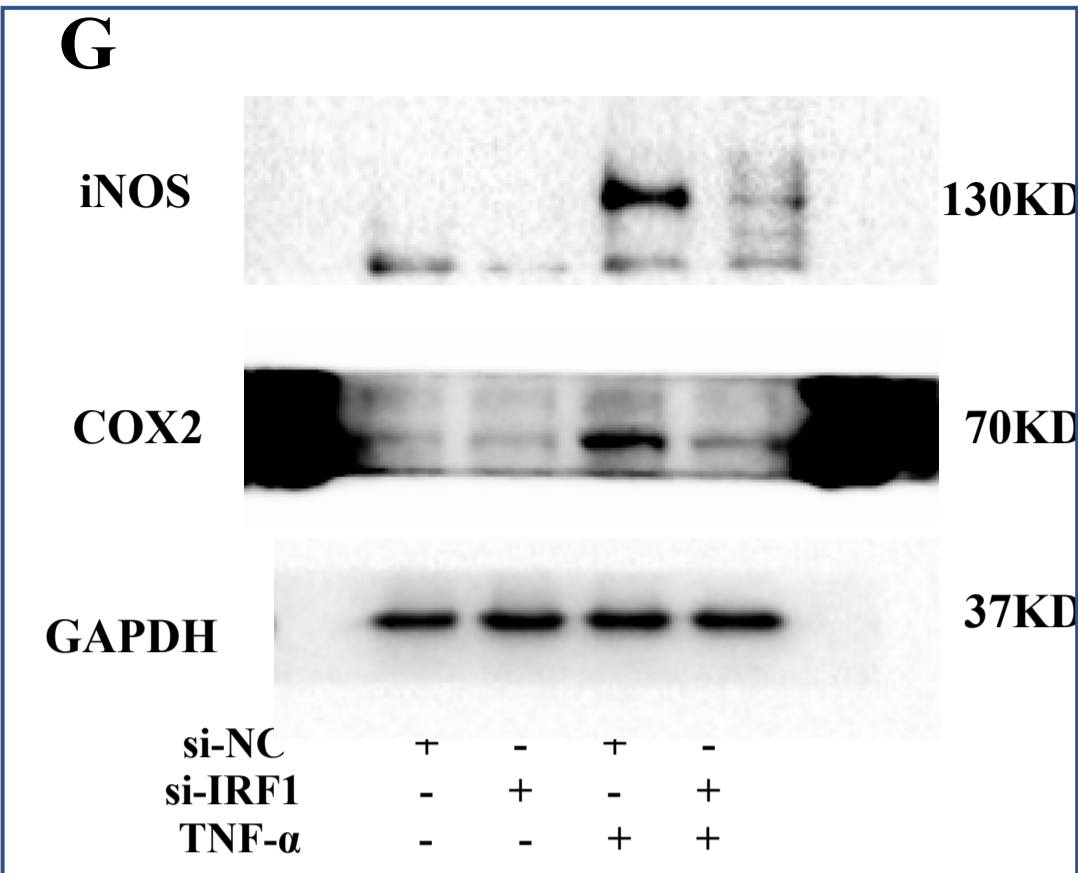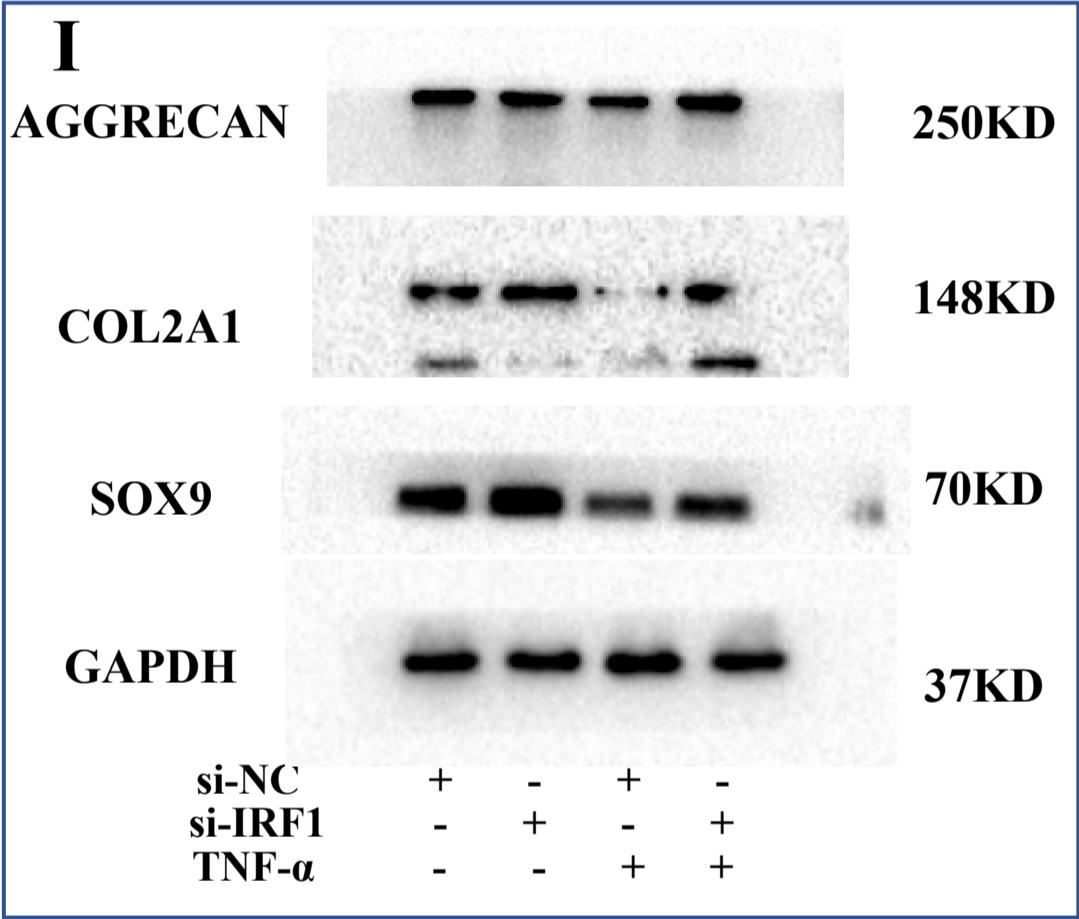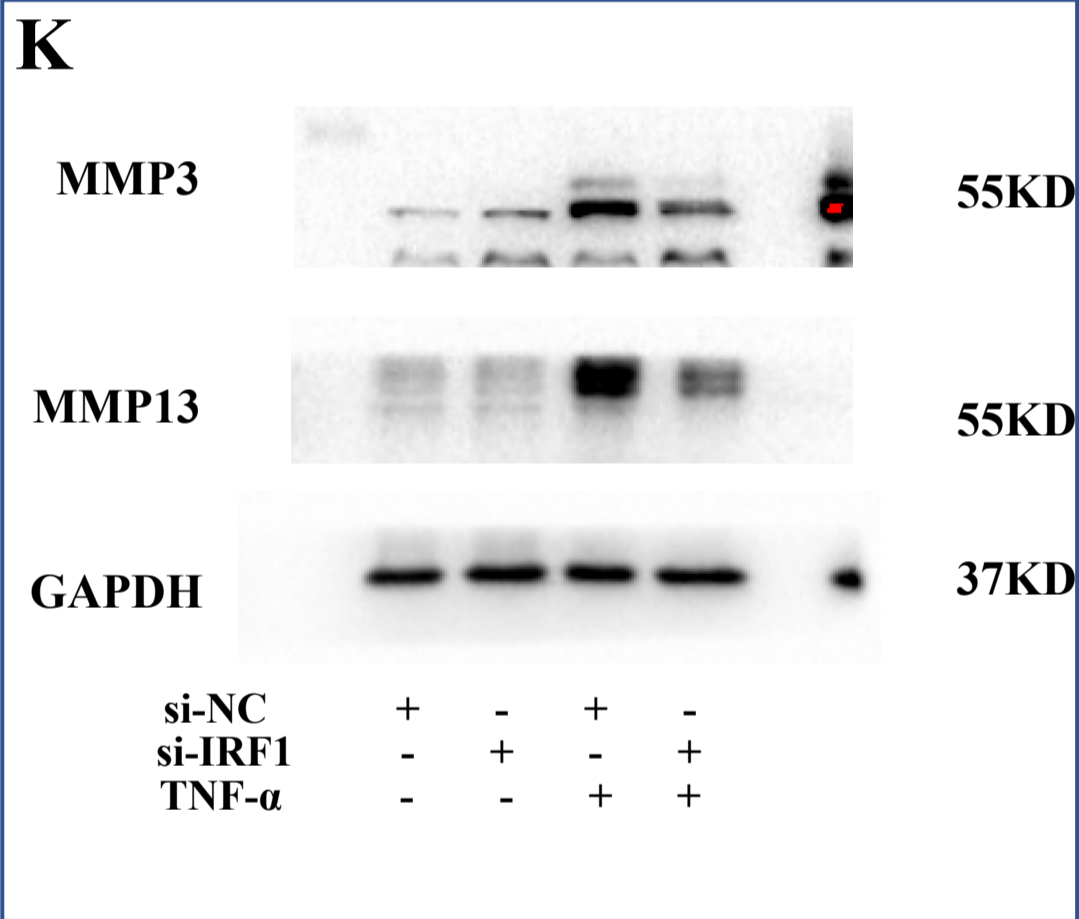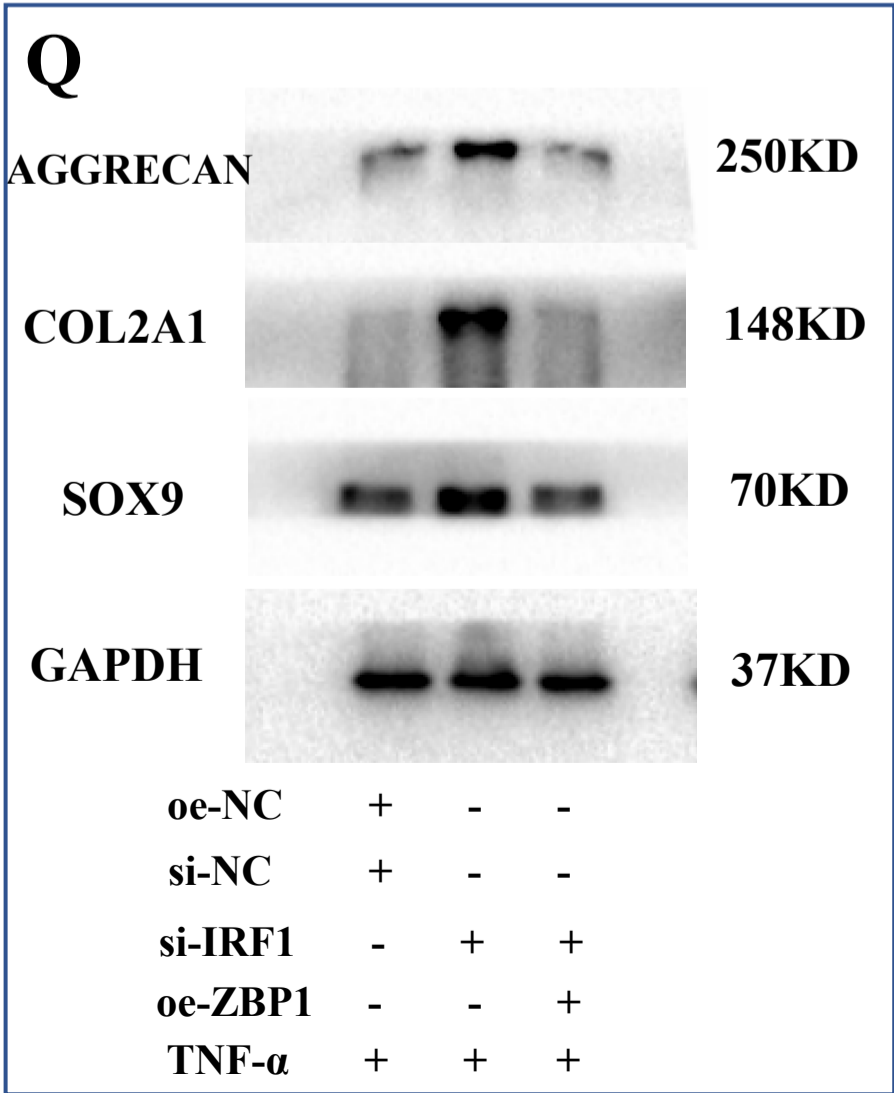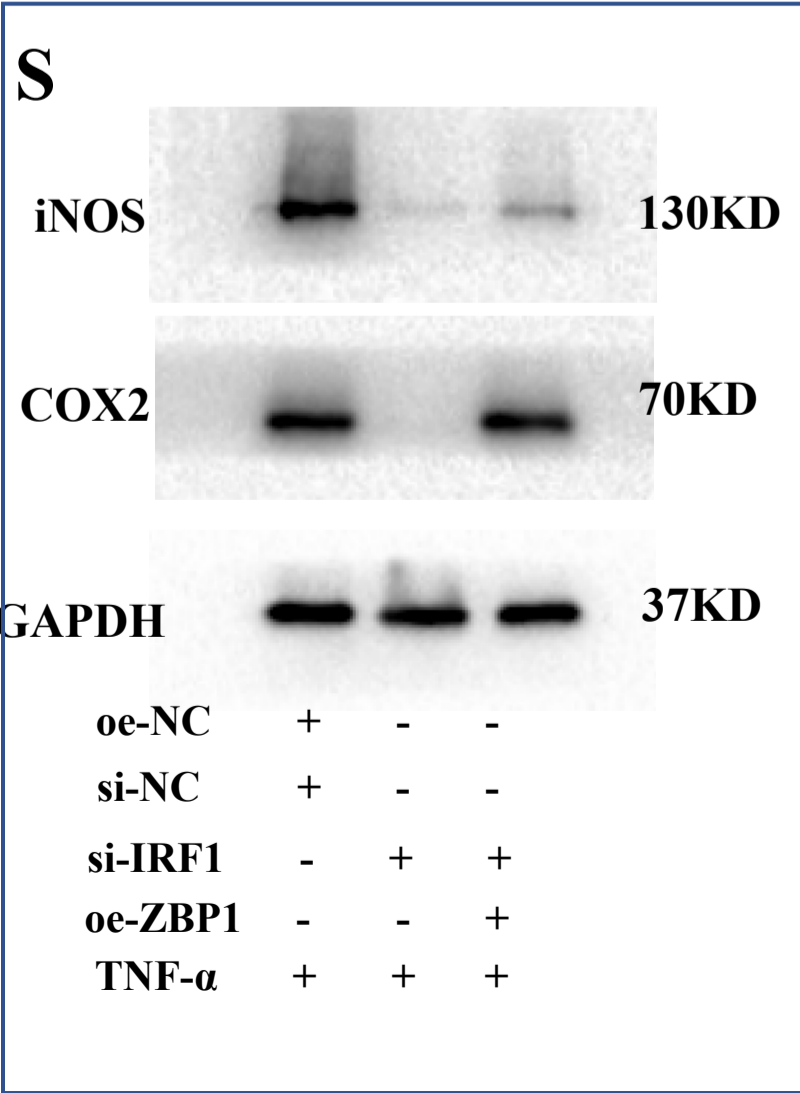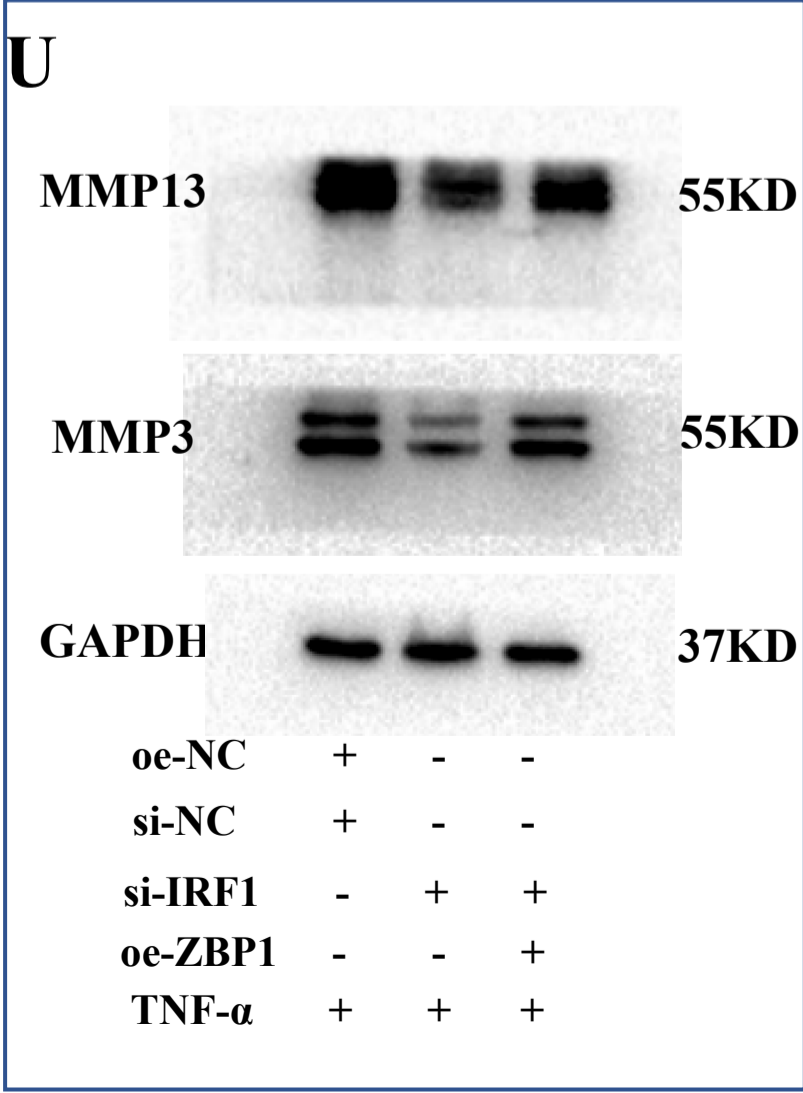

Fig.7

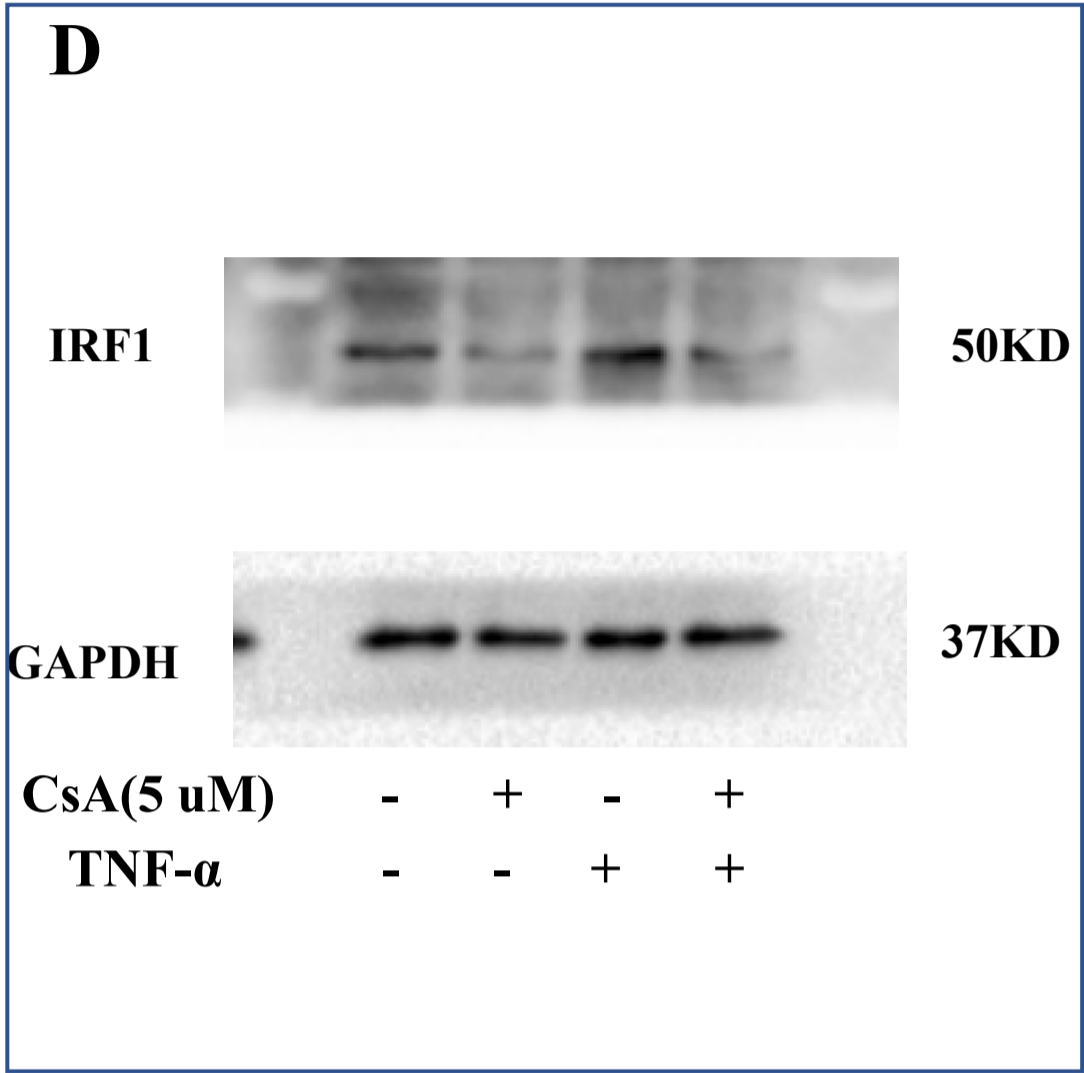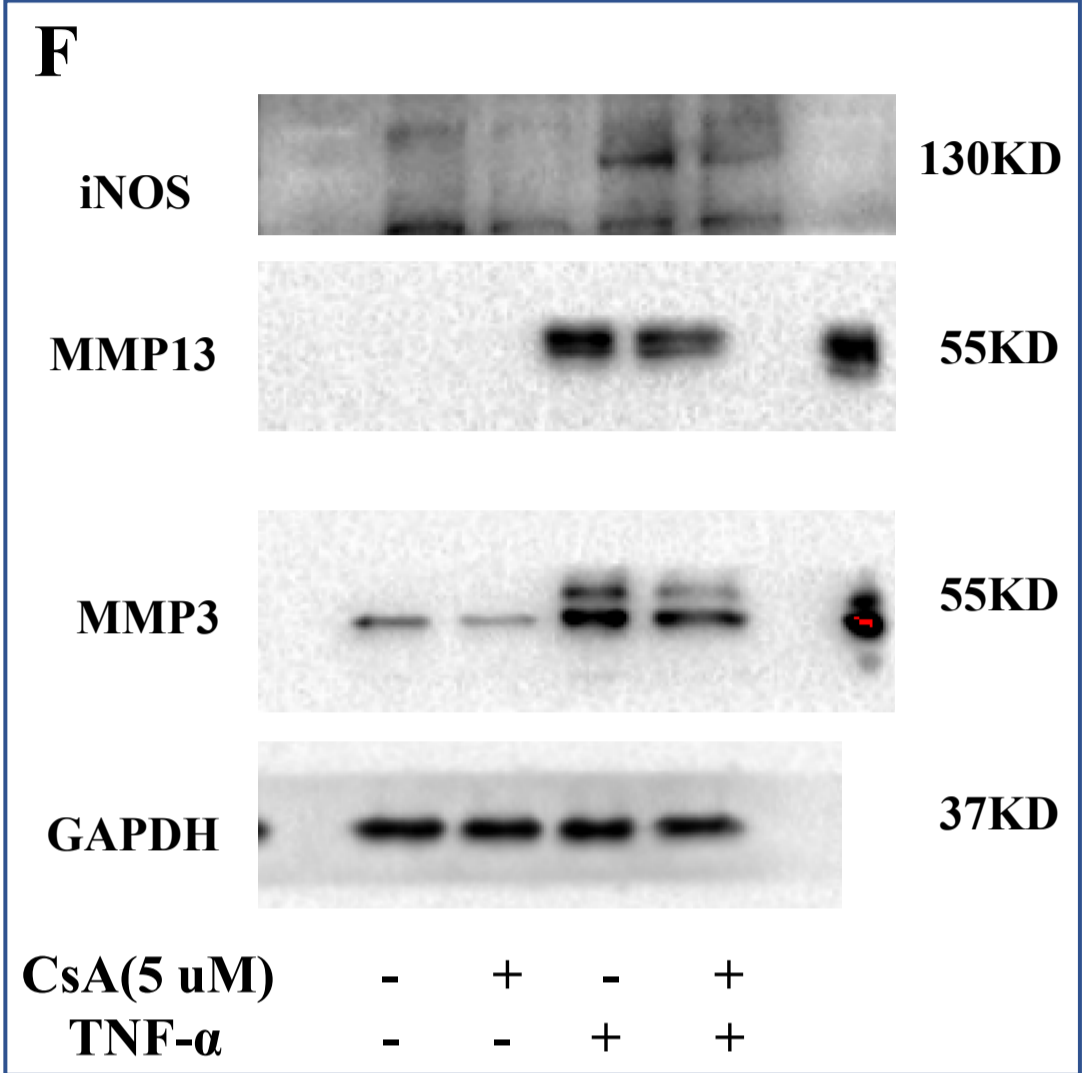

Supplementary Fig.2

A

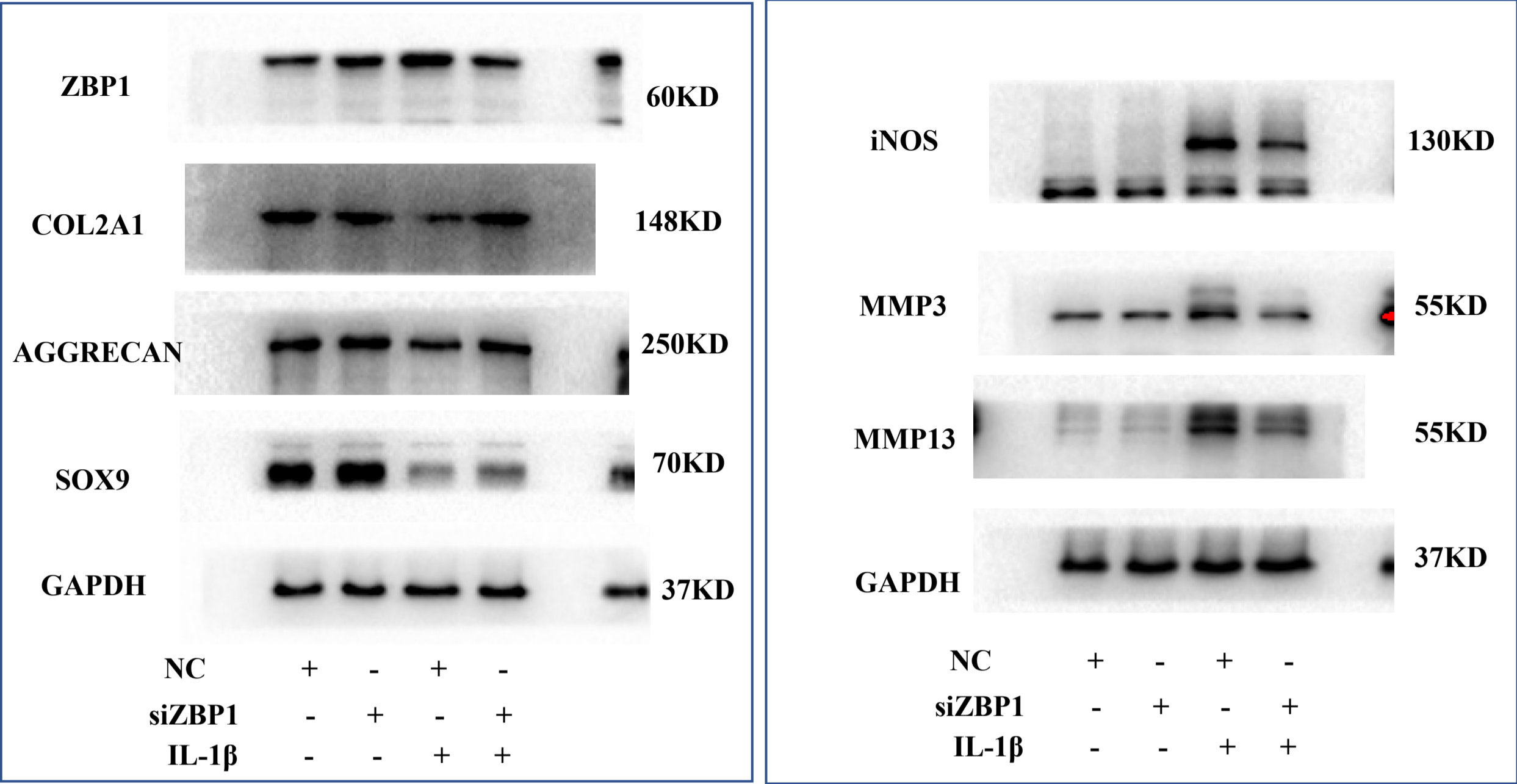

Supplementary Fig.4

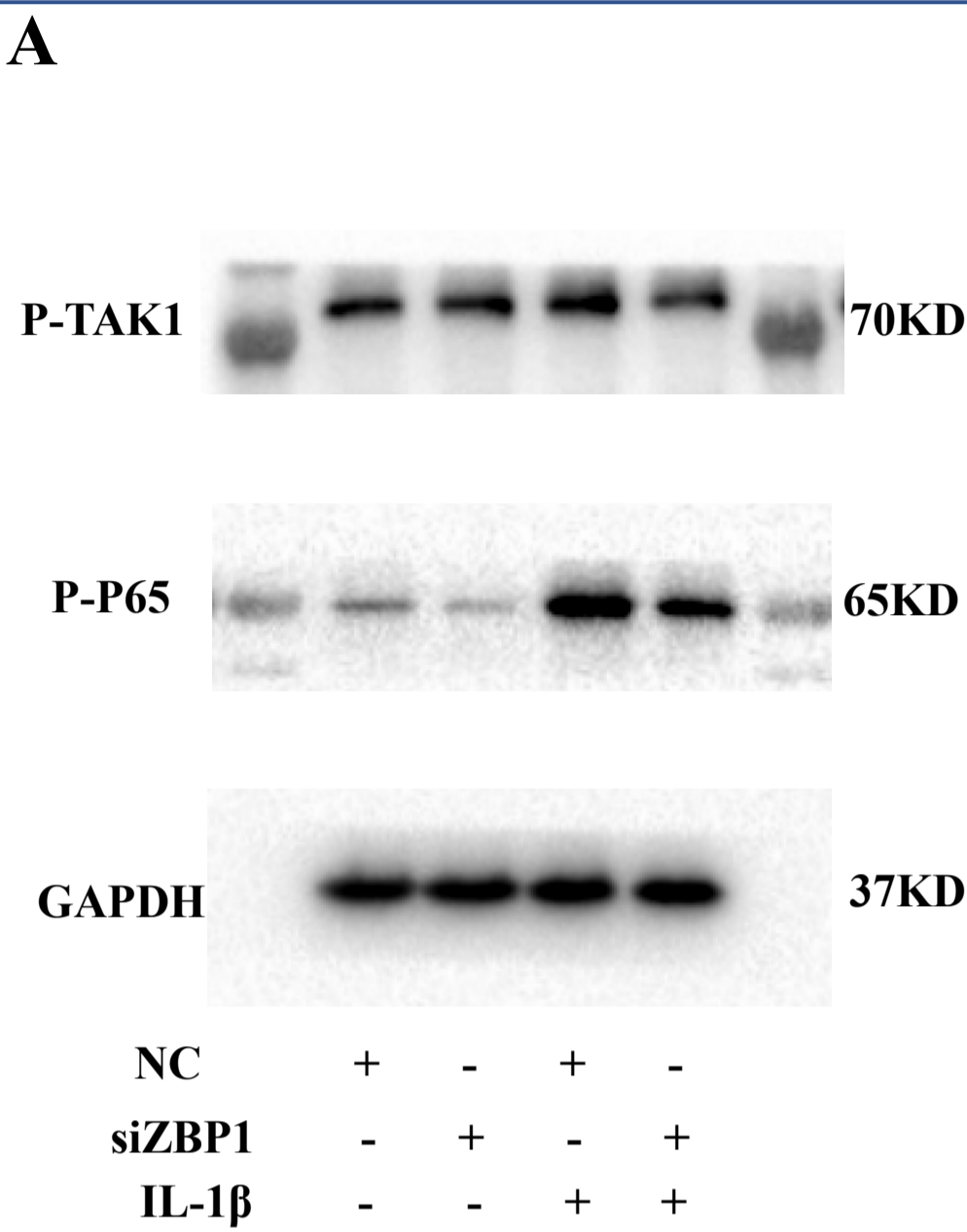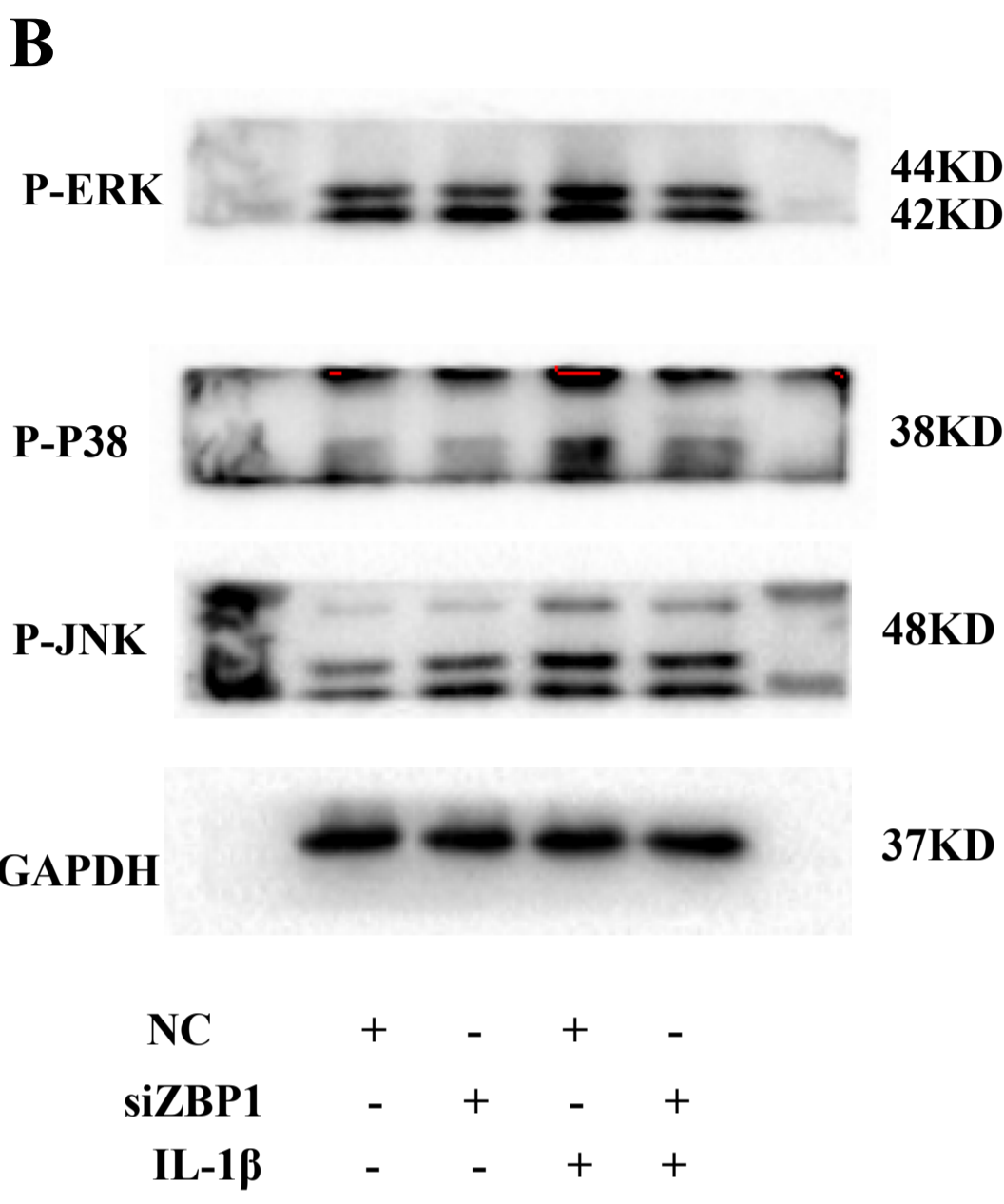

Supplement: Supplementary file 2 — Supplementary Material 2 [file 12964_2024_1744_MOESM2_ESM.pdf]
